# Ultrasound-assisted synthesis of surface state engineered BN-carbon quantum dots for fluoroquinolone sensing: DFT insights^☆^

**DOI:** 10.1016/j.ultsonch.2026.108094

**Published:** 2026-10-02

**Authors:** Duraisamy Elango, Velu Subash, Palaniyappan Jayanthi, Velu Manikandan, Kwang Soup Song

**Affiliations:** a Department of Biomedical Engineering, Kumoh National Institute of Technology, Gumi, South Korea; b High-tech Medical Equipment Research Institute, Kumoh National Institute of Technology, Gumi, South Korea; c Department of Environmental Science, Periyar University, Salem 636011, Tamil Nadu, India

**Keywords:** Carbon quantum dots, Ultrasound-assisted synthesis, Sonochemistry, Fluorescent sensing, Fluoroquinolones

## Abstract

The development of ultrasound-assisted strategies for synthesizing carbon-based nanomaterials has attracted increasing attention owing to the unique physicochemical properties and broad environmental applications. In this study, highly fluorescent surface-state-engineered boron- and nitrogen-co-doped carbon quantum dots (BN-CQDs) were synthesized via an ultrasound-assisted hydrothermal approach using an ultrasonic bath operating at 160 W and a fixed frequency of 37 kHz. The ultrasound-assisted process, together with boron and nitrogen, influenced the physicochemical and optical properties of the resulting BN-CQDs, yielding well-dispersed nanodots with surface-active functional groups and suitable optical properties for the detection of fluoroquinolone antibiotics. The synthesized BN-CQDs were systematically characterized using UV–vis spectroscopy, PL, FTIR, XRD, and XPS. The BN-CQDs exhibited intense blue fluorescence with maximum excitation and emission wavelengths of 380 and 440 nm, respectively, and a high fluorescent quantum yield of 56.8%, highlighting their favorable optical properties for sensing. The fluorescence intensity decreased gradually with increasing concentrations of ciprofloxacin (CIP) and levofloxacin (LVX), primarily attributed to photoinduced electron-transfer and charge-transfer interactions between antibiotic molecules and the surface-active functional groups of the BN-CQDs. Under optimized conditions, the developed fluorescent nanoprobe exhibited a linear response over the concentration range of 0 to 50μmol L^−1^, with low detection limits of 0.286μmol L^−1^ for CIP and 0.320μmol L^−1^ for LVX. Moreover, the sensing platform exhibited excellent selectivity against common metal ions, biomolecules, and interfering antibiotics. Its practical applicability was validated by satisfactory recoveries of 98.0 to 102.0% for CIP and LVX in drinking and tap water samples. DFT calculations provided further insights into the charge-transfer interactions responsible for fluorescence quenching. The ultrasound-assisted synthesis of BN-CQDs provides a promising sonochemical route for developing fluorescent nanoprobes for the sensitive detection of fluoroquinolone antibiotics.

## Highlights

• Ultrasound-assisted BN-CQDs synthesized at 160 W and 37 kHz.
• Ultrasound-assisted synthesis yielded blue-emitting BN-CQDs with 56.8% QY.
• BN-CQDs enabled selective and rapid fluorescence detection of fluoroquinolones.
• Real water samples showed satisfactory recoveries of 98.0 to 102%.

## Graphical abstract

**Figure:**
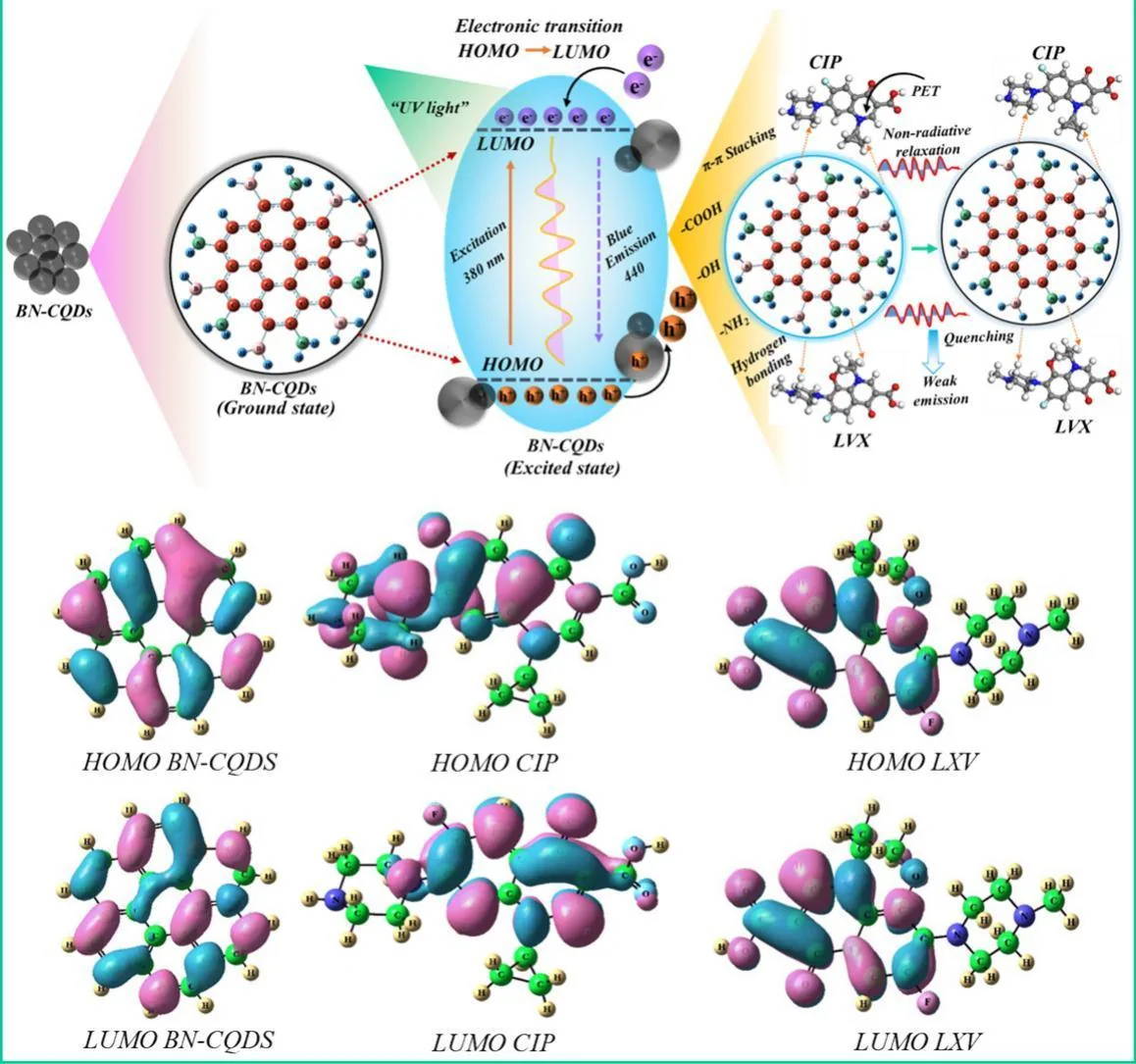

## 1 Introduction

The development of advanced nanomaterials with tailored optical properties has significantly improved the performance of fluorescent-based sensing platforms. Among various nanomaterials, quantum dots have attracted considerable attention owing to their size-dependent fluorescence and tunable emission characteristics. Carbon quantum dots (CQDs), typically below 10 nm in size, have emerged as a promising fluorescent nanomaterial due to their excellent aqueous stability, ease of surface modification, excellent biocompatibility, low intrinsic toxicity, high photostability, and strong photoluminescence [1], [2], [3]. Their optical performance can be further engineered through heteroatom doping and surface- state modulation, which allows fine control over their electronic structure and emission behavior [4]. Owing to these advantages, CQDs have been widely explored in fluorescence sensing, bioimaging, drug delivery, and environmental monitoring applications [5], [6]. Various synthetic approaches have been developed for CQD preparation, including electrochemical, ultrasonication (US), microwave-assisted, solvothermal, and hydrothermal methods [7]. Among these approaches, ultrasound-assisted synthesis has gained increasing attention due to its operational simplicity, economic feasibility, and environmentally friendly nature [8], [9], [10]. The sonochemical effects can improve precursor dispersion, accelerate chemical reactions, influence nucleation, carbonization, and enhance surface functionalization during CQD formation [11], [12]. In particular, US parameters such as frequency, acoustic power, irradiation time, and reaction temperature can affect the physicochemical and photoluminescence properties of CQDs [13], [14]. Therefore, understanding and controlling sonochemical conditions is essential for developing ultrasound-assisted routes toward functional carbon nanomaterials.

The pervasive use of antibiotics has improved the treatment of infectious diseases in both human and veterinary medicine. Among these antibiotics, ciprofloxacin (CIP) and levofloxacin (LVX) are second-generation fluoroquinolone antibiotics that are extensively prescribed owing to their broad-spectrum antimicrobial activity, favorable pharmacokinetic profile, and high therapeutic efficacy [15]. Despite its clinical importance, extensive use and incomplete metabolism of CIP have led to its continuous release into natural ecosystems through hospital effluents, pharmaceutical wastewater, livestock farming, and municipal sewage. Consequently, a measurable concentration of CIP has been reported in surface water, groundwater, soil, food products, and aquatic organisms [16]. Persistent environmental exposure to antibiotics accelerates the development of antibiotic-resistant bacteria and resistance genes, posing a serious threat to ecosystem stability and public health and contributing to life-threatening complications, including kidney damage, allergic reactions, and organ toxicity [17], [18]. These concerns have created an urgent need for analytical platforms capable of sensitive and reliable monitoring of CIP residues in environmental and food matrices.

Conventional analytical techniques, including chromatographic and electrochemical methods, provide excellent sensitivity and quantitative accuracy for CIP detection. However, these approaches generally require sophisticated instrumentation, labor-intensive sample preparation, and relatively long analysis times, limiting their applicability for rapid, decentralized, and on-site monitoring [19]. Fluorescence-based sensing has therefore emerged as an attractive alternative owing to its operational simplicity, rapid response, low cost, and high sensitivity [20]. In particular, CQDs-based fluorescent probes have demonstrated remarkable potential for antibiotic detection by converting specific molecular interactions into measurable optical signals [21]. Several recent studies have reported fluorescent probes for the sensitive detection of CIP and LVX, employing fluorescence quenching, ratiometric fluorescence, and other signal transduction strategies. Sun et al. developed CQDs derived from embryonic chrysanthemums through a green hydrothermal route and used them as a fluorescent probe for CIP detection [22]. These studies demonstrate the potential of fluorescent nanoprobes for rapid fluoroquinolone monitoring while highlighting the importance of probe surface chemistry and molecular interactions in achieving high selectivity and sensitivity [23], [24]. A single quenching pathway governs the fluorescence response in most systems, while the influence of heteroatom-induced surface-state engineering on molecular recognition and fluorescence modulation toward fluoroquinolone remains limited [3]. Therefore, the development of a CQD-based fluorescent nanoprobe with facile synthesis, superior photostability, sensitive detection capability, high selectivity, and well-elucidated sensing mechanisms remains an important research objective.

In this work, we developed a facile ultrasound-assisted hydrothermal strategy for synthesizing surface-state-engineered boron and nitrogen co-doped CQDs (BN-CQDs) for the sensitive detection of CIP and LVX. An ultrasonic bath operated at 160 W and 37 kHz was employed for precursor treatment prior to hydrothermal carbonization. The modulation of the electronic structure by B, N co-doping and abundant surface functional groups yields highly emissive nanodots with excellent aqueous stability and strong affinity toward CIP. Upon interaction with CIP and LVX, the fluorescence of BN-CQDs was efficiently quenched through a mechanism dominated by the inner filter effect, accompanied by photoinduced electron transfer arising from surface-state interactions. Under optimized conditions, the developed nanoprobe exhibits a broad linear response, excellent selectivity toward common antibiotics, rapid fluorescent response, and a low detection limit. The practical applicability of the sensing platform was further demonstrated through the successful determination of CIP and LVX in real water samples with satisfactory recoveries, highlighting its potential for environmental monitoring and food safety analysis.

## 2 Methodology

### 2.1 Experimental reagents

Citric acid (CA), boric acid (BA), urea, glucose, sucrose, Bovine serum albumin (BSA), L-cysteine, glycine, ascorbic acid, ethanol, hydrochloric acid (HCl), sodium hydroxide (NaOH), sodium chloride (NaCl), potassium chloride (KCl), phosphate-buffered saline (PBS), calcium chloride (CaCl_2_), zinc nitrate hexahydrate (Zn (NO_3_)_2_·6H_2_O), aluminium nitrate nonahydrate (Al(NO_3_)_3_·9H_2_O), magnesium sulfate heptahydrate (MgSO_4_·7H_2_O), iron (II) chloride (FeCl_2_), cobalt nitrate hexahydrate (Co (NO_3_)_2_·3H_2_O), nickel (II) nitrate hexahydrate (Ni (NO_3_)_2_·6H_2_O), copper (II) nitrate trihydrate (Cu (NO_3_)_2_·6H_2_O), ciprofloxacin (CIP, 99%), levofloxoacin (LVX, 99%), tetracycline (TC, 99%), oxytetracycline (OTC, 99%), kanamycin (KAN, 99%), amoxicillin (AMO, 99%), sulfamethoxazole (SMZ, 99%), and streptomycin (SM, 99%), were obtained from Merck (South Korea). All the chemicals and reagents were of analytical grade and used as received without further purification. Ultrapure water (18.25 MΩ) was used throughout the experiment. The standard solutions of antibiotics and metal ions were prepared in ultrapure water and stored at 4 °C before use.

### 2.2 Synthesis of BN-CQDS

B, N, co-doped CQDs were synthesized through an ultrasound-assisted hydrothermal approach. In a typical synthesis, 1.0 g of CA, 0.5 g of BA, and 0.5 g of urea were dissolved in 30 mL of ultrapure water under continuous magnetic stirring for 30 min until a clear, homogeneous solution was obtained [25]. The precursor solution was subsequently subjected to US irradiation for 5 h using an ultrasonic bath (dimension: 240 mm × 140 mm × 100 mm) operated at an output power of 160 W and fixed frequency of 37 kHz (JPL Sonic 3MX, China). The sonicated homogeneous solution was transferred to 50 mL Teflon-lined stainless-steel autoclave, sealed tightly, and heated at 180 °C for 12 h. During the hydrothermal reaction, dehydration, polymerization, aromatization, and carbonization of the precursor molecules occurred simultaneously, while boron and nitrogen atoms were incorporated into the carbon framework to generate surface-functionalized BN-CQDS. After completion of the reaction, the autoclave was allowed to cool naturally to room temperature. The resulting dark-yellow suspension was centrifuged at 10,000 rpm for 15 min to remove large carbonaceous particles and insoluble residues. The supernatant was subsequently passed through a 0.22 μM membrane filter to eliminate any remaining particulate impurities. The purified BN-CQDS solution was stored at 4 °C for subsequent characterizations and sensing experiments. A schematic illustration of the ultrasound-assisted hydrothermal synthesis of BN-CQDs is presented in Scheme 1.

**Scheme 1:**
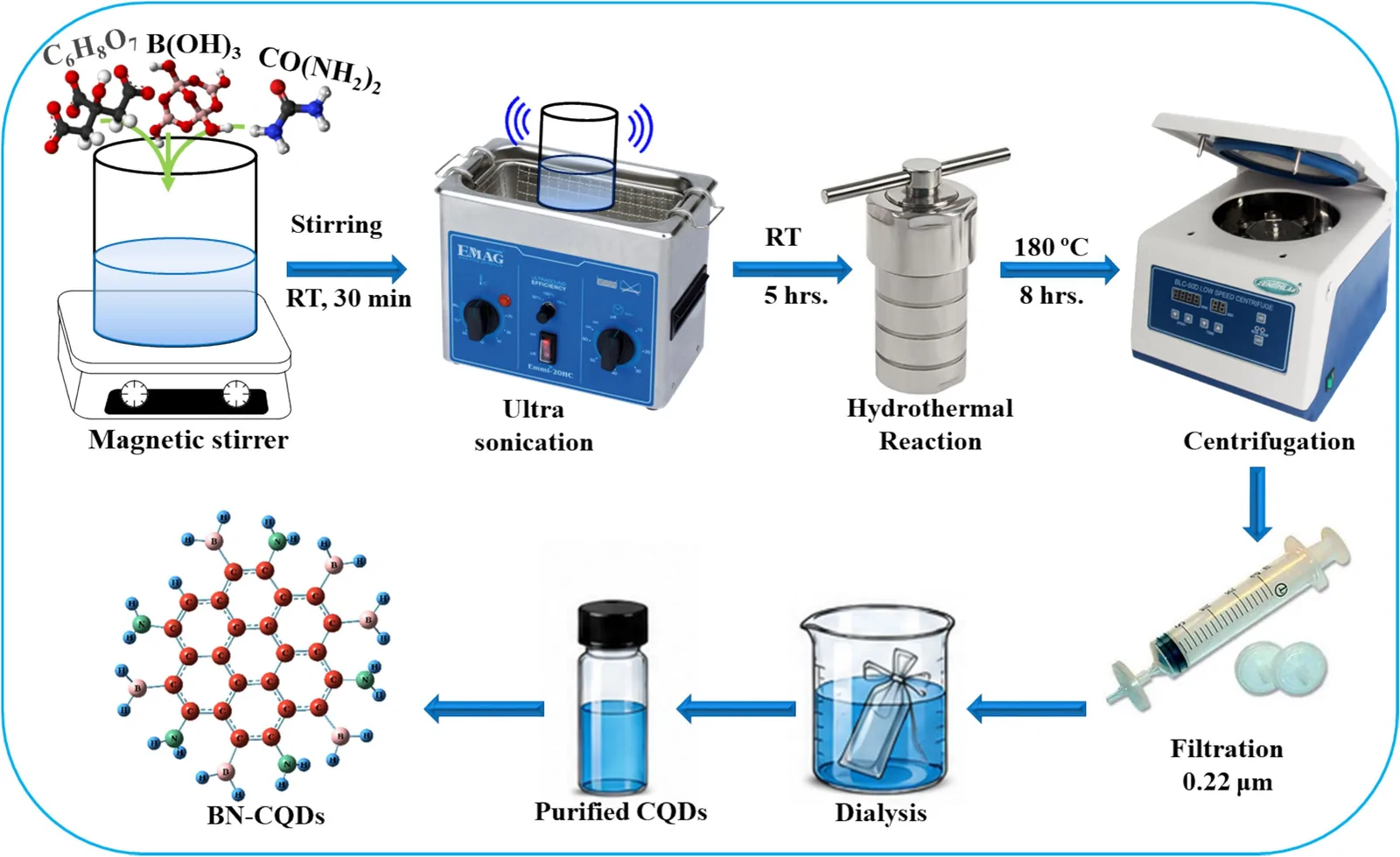
Illustration of the ultrasound-assisted hydrothermal synthesis process of BN-CQDs.

### 2.3 Characterization techniques

The optical properties of BN-CQDs were evaluated using a UV–visible spectrophotometer over the wavelength range of 200–800 nm. Fluorescence excitation and emission spectra were recorded with a fluorescence spectrophotometer to investigate the photoluminescence properties and determine the optimum excitation wavelength. The crystalline characteristics were examined by powder X-ray diffraction (XRD) using a Cu Kα radiation source (λ = 1.5406 Å). XRD patterns were recorded over an appropriate 2θ range of 10°-90° to evaluate the crystallinity and graphitic nature of the carbon nanostructures. The surface functional groups and chemical bonding were investigated using Fourier-transform infrared (FTIR) spectroscopy with the spectral range of 4000–400 cm^−1^. The morphology, particle size distribution, and lattice features of BN-CQDS were characterized by high-resolution transmission electron microscopy (HR-TEM). The surface elemental composition and chemical states of the prepared BN-CQDS were determined by X-ray photoelectron spectroscopy (XPS). Survey and high-resolution spectra were acquired to identify the bonding configurations of C, N, B, and O atoms.

### 2.4 Ultrasonic power measurement

The effective acoustic power of the ultrasonic bath was estimated using a calorimetric method based on the liquid's temperature rise during sonication. The power density was calculated as described in the literature [26]. Briefly, 30 mL of ultrapure water was placed in a flat-bottom glass vessel (7 cm height, 5 cm outer diameter, 4.82 cm inner diameter) and subjected to the same operating conditions used for BN-CQD synthesis. The temperature of the water was recorded at 30 s intervals for 3 min, and the initial temperature rise rate (*dT/dt*) was determined from the slope of the temperature (K) versus time (s) plot. The dissipated ultrasonic power (P) and volumetric power density (D) were calculated according to Equation (1) and Equation (2), respectively.

(1) $$P=\frac{\mathrm{dT}}{\mathrm{dt}}\;Cp\;m$$

(2) $$D=\frac{P}{V}$$

where P is the dissipated ultrasonic power (W), dT/dt is the temperature-rise rate.

(K s^−1^), C_p_ is the specific heat capacity (J g^−1^ K^−1^), and m is the mass of water (g). The volumetric density power D (W dm^−3^) was calculated considering the power (W) and the volume of water (dm^−3^). Results are illustrated in supporting information (Fig. S1 and Table S1).

Detailed quantum yield measurement, stability evaluation tests, along with CIP and LVX detection studies, interference studies, selectivity and real-sample analysis are provided in the Supporting information.

## 3 Result and Discussion

### 3.1 Optical, functional and structural characterization of BN-CQDs

The optical properties of the synthesized BN-CQDs were systematically investigated using UV–visible absorption and PL spectroscopy. The UV–visible absorption spectrum (Fig. 1a) exhibits a prominent absorption band in the UV region, which can be attributed to the π-π* transition of the aromatic C=C bonds and n-π* transition associated with surface functional groups such as C=O, C-N, and heteroatom-induced defect states. The presence of a shoulder peak extending toward the visible region suggests enhanced surface-state contributions induced by B and N heteroatom incorporation, which modify the electronic structure of the carbon core [27]. The photoluminescence behavior demonstrates strong excitation-dependent emission characteristics, a typical feature of quantum-confined carbon nanodots. Under varying excitation wavelengths, the emission spectra exhibit a gradual redshift along with changes in intensity, indicating the presence of multiple emissive trap states and functional-group distribution (Fig. S2). The BN-CQDs exhibited the strongest fluorescence emission at 440 nm upon excitation at 380 nm (Fig. 1b). The maximum emission is observed in the blue region of the visible spectrum, confirming the formation of highly fluorescent CQDs with a narrow size distribution and effective surface passivation. The observed Stokes shift between the excitation and emission peaks suggests efficient radiative recombination via surface-defect-mediated pathways [28]. The co-doping of boron and nitrogen plays a crucial role in tailoring the electronic density and introducing mid-gap states, thereby enhancing the quantum yield and tunability of emission [29]. To further evaluate the contribution of US pretreatment, BN CQDs were also prepared without US pretreatment. The resulting BN-CQDs without US irradiation (BN-CQDs-NS) exhibited differences in UV (Fig. S3a) and PL characteristics compared with the ultrasound-assisted (BN-CQDs-US) sample. The BN-CQDs-NS exhibited excitation/emision maxima at 378/439 nm, whereas BN-CQDs-US exhibited the corresponding maxima at 380/440 nm (Fig. S3b and c), demonstrating that US pretreatment has a measurable influence on the optical characteristics. Acoustic cavitation generated during US irradiation can induce intense microstreaming and enhance mass transfer, facilitating interactions among the carbon, boron, and nitrogen precursors prior to hydrothermal carbonization [14], [30]. Such sonochemical effects can influence precursor transformation and the development of surface states, which are closely associated with the photoluminescence behaviour of CQDs [8]. Recent studies have demonstrated that US processing can regulate the morphology and optical properties of CQDs, highlighting the importance of ultrasound conditions in their synthesis [9], [14]. These optical results confirm the successful formation of BN-CQDs with strong and tunable fluorescence, making them promising candidates for sensing and optoelectronic applications in environmental analytical systems.

**Fig. 1:**
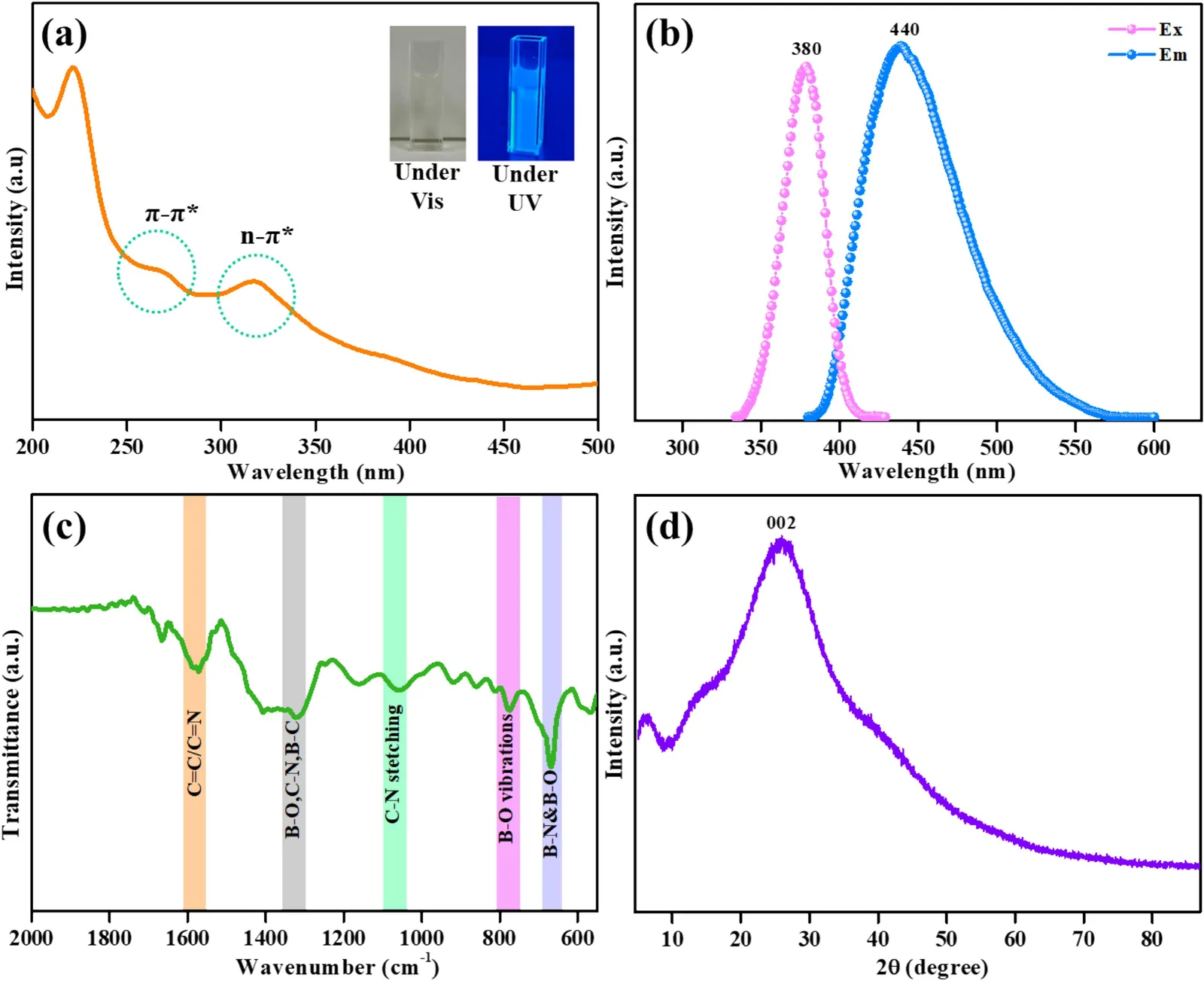
(a) UV–vis adsorption spectra, (b) Photoluminescence Spectra, their excitation and emission spectra recorded at the optimum excitation wavelength of 380 nm, (c) FTIR spectra, and (d) XRD pattern of BN-CQDs.

The successful formation and surface functional groups of BN-CQDs were confirmed by FTIR spectroscopy, which exhibited the presence of various oxygen-, nitrogen-, and boron-containing functional groups on the CQDs surface (Fig. 1c). The characteristic peaks at 1550 cm^−1^ correspond to stretching vibrations of C=C/C=N, indicating the formation of graphitic sp^2^ carbon domains and successful nitrogen incorporation into the carbon framework. The broad band around 1250 cm^−1^ is assigned to the overlapping stretching vibrations of B—O—C, C—N, and B—C bonds, providing strong evidence for successful co-doping of B and N with the CQD surface. Furthermore, the absorption peak near 1050 cm^−1^ is attributed to C-N stretching, confirming the formation of nitrogen-containing functional groups, enhancing the electronic conductivity, and providing active sites for biomolecules immobilization. The band observed around 850 cm^−1^ corresponds to B-O stretching vibrations, indicating boron-oxide-related surface functionalities, while the low-frequency absorption near 650 cm^−1^ is assigned to B-N/B-O vibrations, further confirming the chemical incorporation of B and N into the carbon lattice. The coexistence of these characteristic functional groups demonstrates that B and N atoms were successfully integrated into the CQD framework rather than being physically adsorbed on the surface. The abundant oxygen-containing groups improve the hydrophilicity and colloidal stability of the CQDs, whereas nitrogen doping enhances electrical conductivity and electron-donating ability, and B doping introduces electron-deficient sites that promote charge polarization and electron transfer [31], [32]. The synergistic effect of B and N co-doping increases the defect density, modifies the electronic structure, and creates abundant active sites, thereby improving the optical, electrochemical, and catalytic properties of the BN-CQDs [33]. These surface functionalities and heteroatom dopants are expected to facilitate efficient charge transport, strong biomolecular interactions, and enhanced sensing performance, making the synthesized BN-CQDs highly promising materials for fluorescence applications.

The crystallographic structure of the synthesized BN-CQDs was investigated by XRD analysis, and the corresponding diffraction pattern is illustrated in Fig. 1d. The XRD profile exhibits a broad diffraction peak at 2θ about 26°, corresponding to the (002) plane of graphitic carbon. The calculated interlayer spacing (d_002_) using Bragg's law is about 0.342 nm, which is aligned with the characteristic interlayer distance of graphitic carbon (∼0.34 nm). The broad nature of the (002) reflection indicates that the synthesized BN-CQDs possess a nanocrystalline graphitic structure with a significant amorphous component, which is a common feature of carbon quantum dots synthesized through hydrothermal carbonization. The incorporation of B and N atoms into the carbon lattice induces lattice distortion and disrupts the long-range graphitic ordering because of the differences in atomic size and electronegativity between carbon and the dopant atoms. Such heteroatom incorporation increases the defect density and creates additional electronic states, which are beneficial for improving electrical conductivity, charge transfer kinetics, and surface reactivity [27], [34]. Notably, no additional diffraction peaks corresponding to crystalline boron oxide and other impurity phases were detected, indicating that the B and N atoms were successfully incorporated into the CQD framework rather than forming separate crystalline phases. The partially graphitized structure combined with abundant defect sites is highly advantageous for fluorescence sensing because it provides efficient electron transport pathways and numerous active sites for analyte adsorption and electron transfer. To further evaluate the role of US pretreatment, the XRD pattern of BN-CQDs prepared without US irradiation was compared with BN-CQDs-US (Fig. S3d). Both samples exhibited a broad peak at 26°. Notably, BN-CQDs-US showed a broader and less intense peak than BN-CQDs-NS, indicating reduced graphitic ordering and a more disordered carbon framework. This structural feature may provide additional accessible sites for analyte interaction, demonstrating the beneficial influence of US pretreatment on the structural characteristics of BN-CQDs.

### 3.2 HR-TEM investigation of BN-CQDs

The morphological, microstructural characteristics, and size distribution of the synthesized BN-CQDs were systematically investigated using HR-TEM and fast Fourier transform (FFT) analysis, as illustrated in Fig. 2a–d. The low-magnification TEM images (Fig. 2a and b) reveal that the synthesized BN-CQDs are uniformly dispersed quasi-spherical nanoparticles without noticeable agglomeration, indicating that the hydrothermal synthesis produced highly stable nanodots with excellent colloidal dispersibility. The absence of significant aggregation can be attributed to the abundant oxygen, boron, and nitrogen-containing surface functional groups, which generate electrostatic repulsion and steric stabilization among individual quantum dots. The particles exhibit a narrow size distribution with excellent monodispersity, demonstrating the effectiveness of heteroatom-assisted nucleation and controlled carbonization during synthesis. Importantly, the FFT results (Fig. 2c) are correlated with XRD analysis, which exhibited a broad diffraction peak centered at 2θ about 26°, corresponding to the (002) plane of graphitic carbon with an interlayer spacing of about 0.342 nm. While the XRD pattern demonstrates the existence of graphitic carbon through the broad (002) reflection, the FFT pattern provides direct nanoscale evidence for the presence of localized graphitic domains within individual carbon quantum dots. The broad diffraction peak observed in XRD and the diffuse ring observed in FFT collectively indicate that the synthesized BN-CQDs possess a partially graphitized nanocrystalline structure rather than a highly ordered graphite lattice. This consistency between reciprocal-space information obtained from FFT and bulk crystallographic information obtained from XRD strongly confirms the formation of defect-rich graphitic quantum dots. Furthermore, the absence of diffraction features corresponding to boron oxide and boron nitride or other impurity phases in both FFT and XRD analyses suggests that boron and nitrogen atoms are uniformly incorporated into the carbon framework rather than forming separate crystalline domains.

**Fig. 2:**
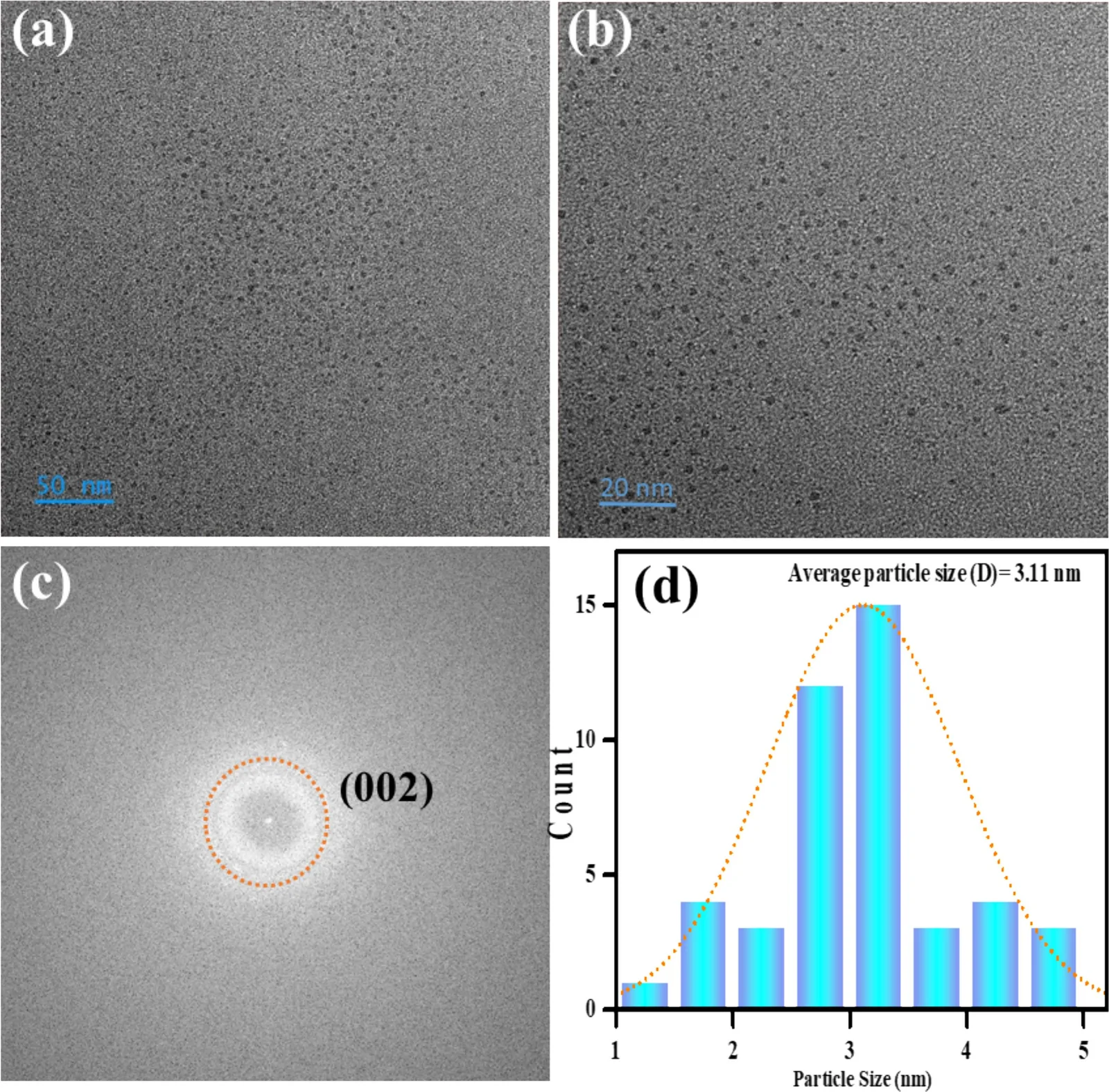
Representative TEM images (a, b), FFT patterns (c), and the particle size distribution (d) of BN-CQDs.

Particle size analysis (Fig. 2d) further confirms the formation of ultra-small quantum dots with diameters ranging from 1.2 to 5.0 nm, while the calculated average particle diameter is 3.11 nm. For comparison, BN-CQDs prepared without US pretreatment exhibited a larger particle size of 4.59 nm with relatively non-uniform morphology, whereas US pretreatment resulted in smaller and more uniformly distributed BN-CQDs (Fig. S4). The narrow particle size distribution indicates uniform nucleation and growth throughout the hydrothermal reaction. Such ultra-small dimensions are one of the defining characteristics of CQDs and are essential for the index of strong quantum confinement and edge effects, which directly influence their optical and electrochemical properties [35]. The high surface-to-volume ratio associated with the nanoscale dimensions also provides abundant active surface sites, facilitating rapid electron transfer and efficient interaction with analyte molecules during the sensing process [36]. Consequently, the synthesized BN-CQDs possess structural and electronic characteristics that are highly favorable for high-performance fluorescence sensing applications.

### 3.3 XPS analysis of BN-CQDs

The surface elemental composition and chemical states of the synthesized BN-CQDs were investigated by XPS analysis, and the corresponding survey spectrum is provided in Fig. S5. The XPS survey spectrum exhibits four distinct characteristic peaks at 191 eV, 285 eV, 399 eV, and 531 eV, which are assigned to B 1s, C 1s, N 1s, and O 1s, respectively. The simultaneous presence of these characteristic signals confirms the successful incorporation of B and N into the CQDs framework together with abundant oxygen-containing surface functionalities. Notably, no additional peaks corresponding to metallic impurities or secondary crystalline phases were detected. Indicating the purity of the synthesized BN-CQDs. The dominant C 1s peak originates from the graphitic carbon backbone, while the intense O 1s demonstrates the presence of oxygen-containing functional groups such as hydroxyl, carbonyl, and epoxy species on the CQDs' surface. The oxygen functionalities improve the hydrophilicity, colloidal stability, and chemical reactivity of the quantum dots while providing abundant sites for subsequent surface functionalization and biomolecule immobilization [37]. The appearance of the N 1s peaks confirms the successful nitrogen doping within the carbon lattice, where nitrogen atoms are incorporated as pyridinic, pyrrolic, or graphitic nitrogen species. Such nitrogen incorporation increases the electron density, enhances electrical conductivity, introduces defect sites, and promotes rapid electron transfer kinetics. Similarly, the presence of the B 1s peak verifies the successful incorporation of boron atoms into the carbon framework rather than their physical adsorption on the surface. Owing to its electron-deficient nature, boron induces charge redistribution within the graphitic lattice, creating positively polarized active sites that facilitate charge transfer and improve catalytic activity [38]. The coexistence of carbon, oxygen, nitrogen, and boron confirms the successful formation of BN-CQDs with abundant heteroatom-functionalized surfaces, consistent with the FTIR results, which revealed the presence of C—N, C=N, B—C, B—O, and B—N bonds. These XPS results provide complementary evidence that boron and nitrogen atoms are successfully integrated into the carbon quantum dot framework, producing a defect-rich graphitic nature with abundant surface functional groups. Such synergistic structural architecture is expected to enhance electrical conductivity, accelerate interfacial electron transfer, increase the density of electrochemically active sites, and improve the adsorption affinity toward target analytes, thereby making the synthesized BN-CQDs highly promising materials for advanced fluorescence sensing applications.

To further elucidate the surface chemical composition and bonding configuration of the synthesized BN-CQDs, high-resolution XPS analysis was performed, and the deconvoluted spectra of C 1s, O 1s, B 1s, and N 1s are presented in Fig. 3(a–d). The high-resolution C 1s spectrum (Fig. 3a) was deconvoluted into several components corresponding to different carbon chemical environments. The dominant component is associated with the C-C/C=C groups, confirming the presence of a graphitic sp^2^–hybridized carbon framework. The appearance of the C-N group provides direct evidence for the successful incorporation of nitrogen into the carbon lattice, while C-O and C=O species indicate the presence of abundant oxygen-containing surface functional groups generated during the hydrothermal carbonization process. These oxygen functionalities enhance the hydrophilicity, colloidal stability, and chemical reactivity of the BN-CQDs and provide active sites for subsequent biomolecular immobilization. The coexistence of graphitic carbon and oxygenated functional groups suggests that the synthesized CQDs possess a conductive carbon core surrounded by chemically active functionalities. The high-resolution O 1s spectrum (Fig. 3b) indicates the presence of oxygen containing surface functionalities associated with C=O and C-O, respectively. These oxygen-containing species contribute to the surface chemitry and hydrophilic nature of the BN-CQDs. Furthermore, these oxygen functionalities contribute to defect generation and surface-state formation, thereby facilitating efficient charge transfer and enhancing fluorescence emission through surface-state modulation. Similarly, the N 1s spectrum (Fig. 3c) exhibits two deconvoluted peaks located at 398.17 eV and 399.56 eV, which are assigned to C-N and N-H species, respectively. The dominant C-N component confirms that nitrogen atoms are incorporated into the graphitic carbon framework rather than physically adsorbed on the surface. Nitrogen incorporation modifies the electronic structure of carbon by increasing electron density and generating localized defect sites, thereby improving electrical conductivity and accelerating interfacial electron transfer kinetics [39]. The presence of N-H species further indicates the existence of nitrogen-containing surface functional groups, which can enhance hydrophilicity and provide additional active sites for molecular recognition and biomolecule attachment. Similarly, the B 1s spectrum (Fig. 3d) was deconvoluted into two characteristic peaks at 190.03 eV and 190.77 eV, corresponding to B—C and B—O bonding configurations, respectively. The presence of B—C bonds demonstrates that boron atoms are successfully incorporated into the carbon lattice, while the B-O component indicates partial bonding of boron with surface oxygen atoms, forming stable boron-oxygen functionalities. Because boron possesses lower electronegativity than carbon, its incorporation creates electron-deficient centers within the graphitic framework, resulting in charge redistribution and enhanced polarization of neighboring carbon atoms [40]. These positively polarized active sites significantly facilitate electron transfer and strengthen interactions with analyte molecules. These structural and functional characteristics make the synthesized BN-CQDs highly promising nanomaterials for fluorescence sensing applications.

**Fig. 3:**
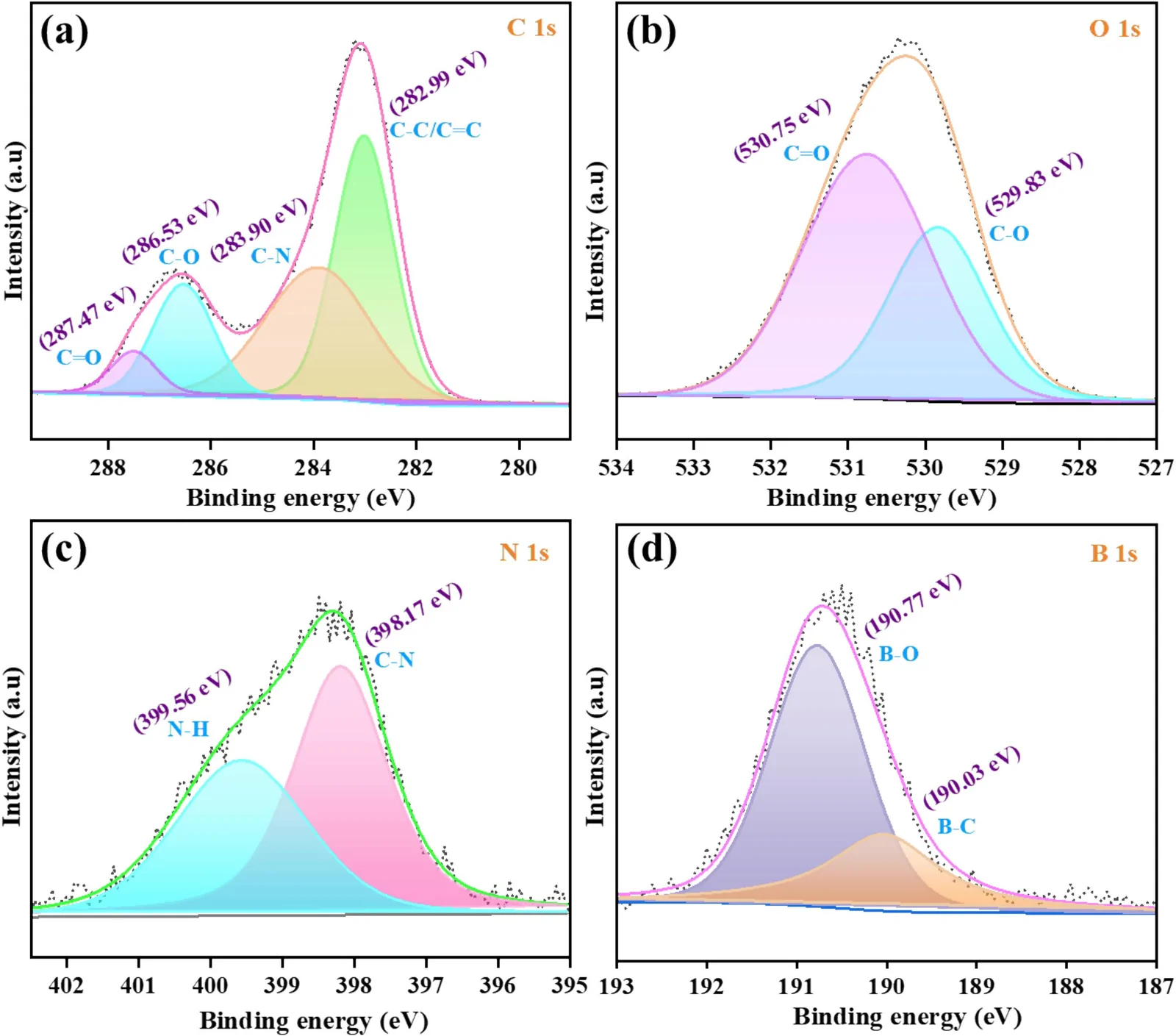
XPS spectrum of BN-CQDs: (a) high-resolution spectrum of C 1s, (b) high-resolution spectrum of O 1s, (c) high-resolution spectrum of N 1s and (d) high-resolution spectrum of B 1s.

### 3.4 Fluorescence quantum yield of BN-CQDs

The fluorescence quantum yield (QY) of the synthesized BN-CQDs was determined using the comparative method with quinine sulfate as a standard fluorophore under identical experimental conditions. The BN-CQDs-US exhibited a QY of 56.8%, whereas the BN-CQDs-NS exhibited a QY of 47.96%, respectively. Thus, ultrasonic pretreatment resulted in an 8.84% increase in the fluoresecnce quantum yield. This value falls within the range commonly reported for heteroatom-doped CQDs synthesized via hydrothermal routes, where QY generally varies between 20 and 60%, depending on the precursor chemistry, degree of graphitization, surface passivation, and heteroatom concentration. The comparative fluorescence QY values of the present BN-CQDs-NS and BN-CQDs-US and previously reported CQDs are summarized in Table S2. The enhanced fluorescence efficiency of the BN-CQDs is primarily attributed to the synergistic electronic effects of boron and nitrogen co-doping. Nitrogen atoms introduce electron-rich localized states within the conjugated carbon framework, increasing electron density and promoting radiative electron-hole recombination, whereas boron atoms, owing to their electron- deficient nature, generate positively polarized centers that modify the electronic band structure and facilitate charge redistribution [41]. The ultra-small particle size of CQDs also contributes to strong quantum confinement, surface-state emission, and hetero-atom-induced electronic modulation, producing highly emissive BN-CQDs with excellent optical performance.

### 3.5 Optimization of sensing conditions and stability of BN-CQDs

The sensing conditions were systematically optimized by evaluating the pH, incubation time, and probe concentration on the fluorescence response. The BN-CQDs showed stable fluorescence at pH 8, which was therefore selected as the working condition (Fig. S6a). The fluorescence response reached a maximum and became stable after 15 min of incubation time and selected as the optimum incubation time (Fig. S6b). The evaluation of different probe concentrations identified 0.1 mg mL^−1^ as the optimum probe concentration for subsequent sensing measurements (Fig. S6c). The practical applicability of fluorescent carbon quantum dots largely depends on their physicochemical stability under different environmental conditions. Therefore, the fluorescence stability of synthesized BN-CQDs was systematically investigated by evaluating the effects of solution pH, ionic strength, long-term storage, and continuous UV irradiation, as presented in Fig. 4(a–d).

**Fig. 4:**
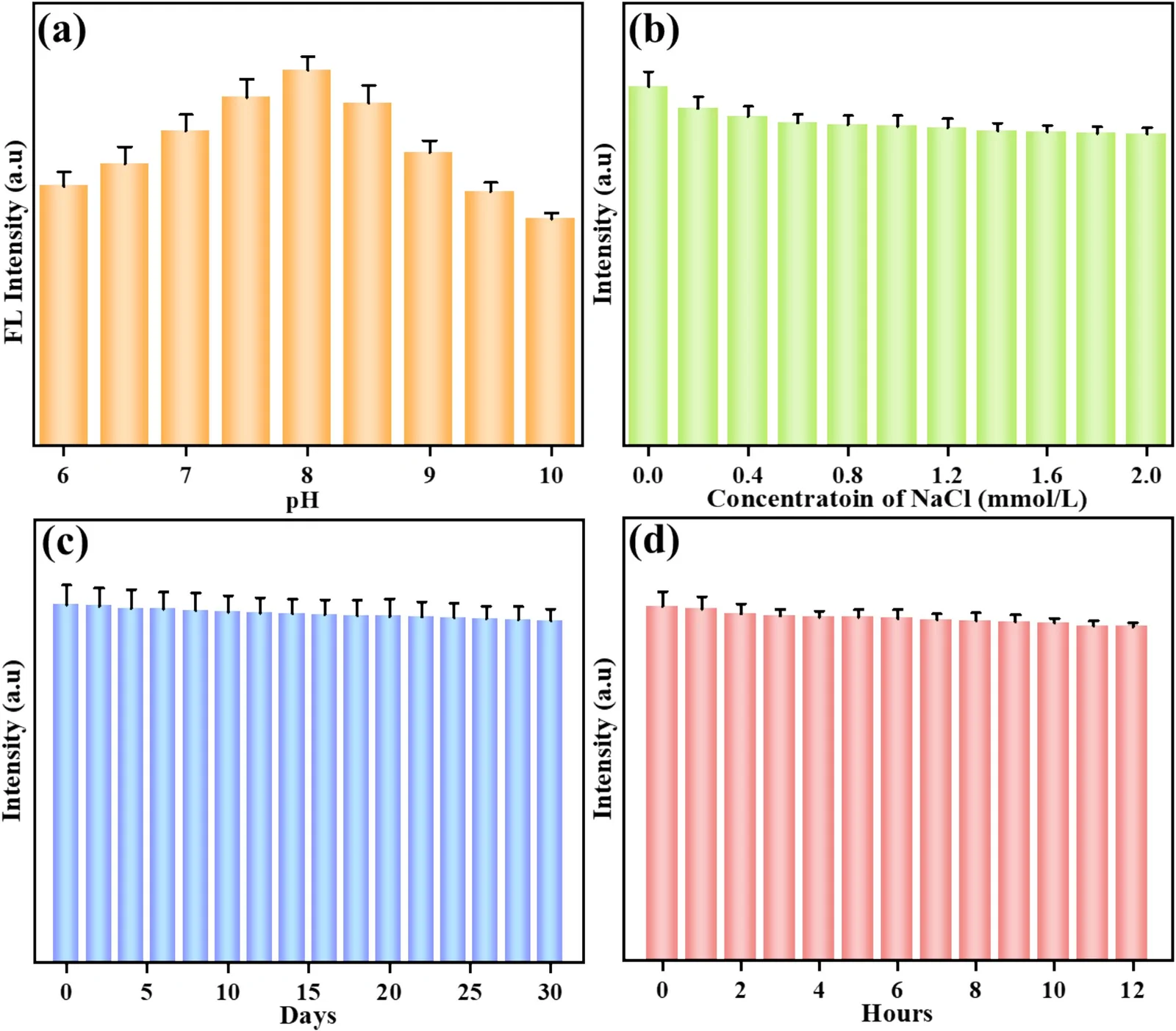
Stability evaluation of the synthesized BN-CQDs under different experimental conditions: (a) effect of solution pH, (b) effect of ionic strength at different NaCl concentrations, (c) storage stability after different storage periods, and (d) photostability under continuous UV irradiation. The fluorescence intensity was measured under identical experimental conditions and the error bars represent the mean ± SD (n = 3).

#### 3.5.1 Effect of pH

The fluorescence response of the BN-CQDs was first evaluated over a broad pH range (6–10) (Fig. 4a). The emission intensity gradually increased from pH 6–8, reaching its maximum at pH 8, followed by a noticeable decrease under alkaline conditions (pH > 8). The enhanced fluorescence under neutral to slightly alkaline conditions indicates that the surface functional groups of BN-CQDs remain highly stable and are favorably protonated/deprotonated, minimizing non-radiative recombination pathways. The fluorescence reduction observed at higher pH values is attributed to excessive deprotonation of oxygen- and nitrogen-containing surface groups, including hydroxyl, carboxyl, amino, and alterations in the electronic structure of the surface states can introduce additional non-radiative relaxation pathways, thereby decreasing fluorescence intensity. Conversely, under mild acidic conditions, partial protonation of these surface groups may also perturb the emissive surface defects, resulting in lower fluorescence. Relatively stable fluorescence behavior in throughout the investigated pH range, demonstrating the excellent chemical robustness of the BN-CQDs toward moderate pH variations commonly encountered in environmental and biological samples [42]. The highest fluorescence stability intensity at pH 8 further confirms that this pH provides the most favorable surface electronic environment for radiative recombination and was therefore selected for optimum operating conditions for subsequent CIP and LVX sensing experiments.

#### 3.5.2 Effect of ionic strength

The influence of ionic strength on fluorescence stability was investigated by introducing NaCl concentrations ranging from 0 to 2 mmol L^−1^ (Fig. 4b). The fluorescence intensity remained nearly unchanged throughout the entire concentration range, exhibiting only negligible fluctuations. The excellent salt tolerance indicates that electrolyte-induced screening effects minimally affect the photoluminescent properties of the BN-CQDs. High concentrations of Na^+^ and Cl^−^ ions generally compress the electrical double layer surrounding nanoparticles, which often promotes aggregation and fluorescence quenching in conventional nanomaterials. However, no significant fluorescence loss was observed for the synthesized BN-CQDs, suggesting excellent colloidal stability [25], [42]. This outstanding stability can be attributed to the abundant hydrophilic surface functional groups introduced during B and N co-doping, which provide strong hydration and steric stabilization. In addition, the dopant-induced modification of the carbon lattice enhances charge distribution and prevents particle agglomeration even under high ionic strength conditions.

#### 3.5.3 Storage stability

Long-term is an essential parameter for evaluating the practical applicability of fluorescent nanoprobes. As illustrated in Fig. 4c, the fluorescence of the BN-CQDs exhibited a slight decrease during 30 days of storage at room temperature. More than 95% of the initial fluorescence intensity was retained after 30 days, indicating excellent photochemical and colloidal stability during prolonged storage. The minimal fluorescence decay suggests that the carbon core and surface passivation layers remain structurally intact without significant oxidation, aggregation, or degradation. The remarkable storage stability can be attributed to the highly graphitized carbon framework together with effective surface passivation by oxygen, nitrogen, and boron-containing functional groups. These excellent shelf-life demonstrate that the synthesized BN-CQDs can be prepared in advance, stored for extended periods, and used without requiring additional purification or regeneration, significantly enhancing their practicality for routine fluorescence sensing.

#### 3.5.4 Photostability under continuous UV irradiation

Photostability is another critical parameter for fluorescence-based sensing because prolonged UV excitation often causes irreversible photobleaching in conventional fluorophores. Therefore, the fluorescence stability of the BN-CQDs under continuous UV irradiation was evaluated for 12 hrs (Fig. 4d). Only a marginal reduction in fluorescence intensity was observed during the continuous exposure, with the BN-CQDs retaining 96% of their initial emission intensity after 12 hrs. This negligible fluorescence loss demonstrates exceptional resistance to photobleaching. The superior photostability originates from the highly conjugated sp^2^ carbon framework and the effective surface defect engineering achieved through boron and nitrogen co-doping. The heteroatom dopants stabilize the emissive surface states and suppress photo-induced oxidation, thereby minimizing irreversible destruction of fluorescence centers during prolonged irradiation. Compared with conventional organic fluorescent dyes, which often experience rapid photobleaching under continuous excitation, the synthesized BN-CQDs maintain highly stable fluorescence, making them particularly suitable for long-term fluorescence sensing and repeated analytical measurements.

### 3.6 Anti-interference performance of BN-CQDs

The anti-interference capability of synthesized BN-CQDs was systematically investigated by monitoring their fluorescence response in the presence of various metal ions and biologically relevant molecules that are frequently encountered in environmental and biological samples (Fig. 5). The fluorescence intensity remained nearly constant after the addition of K^+^, Na^+^, Ca^2+^, Mg^2+^, Al^3+^, Fe^2+^, Co^2+^, Ni^2+^, Cu^2+^, Zn^2+^, glucose, sucrose, BSA, L-cysteine, glycine, and ascorbic acid, indicating that these species do not significantly influence the emissive properties of the BN-CQDs. The fluorescence variation for most interferents was within the experimental error, confirming the excellent photostability and chemical robustness of the synthesized nanodots. A comparatively larger decrease was observed only in the presence of Fe^2+^, while Cu^2+^ produced moderate fluorescence attenuation. This behavior is consistent with previous reports on heterostructured CQDs, where Fe^2+^ and Cu^2+^ exhibit higher binding affinity toward oxygen- and nitrogen-containing functional groups through coordination interactions. The strong Lewis acidity of Fe^2+^ enables complex formation with surface hydroxyl, carbonyl, and carboxyl groups, facilitating photoinduced electron transfer (PET) or ligand-to-metal charge transfer (LMCT), which introduces additional non-radiative decay pathways and partially quenches fluorescence [42]. Likewise, Cu^2+^, owing to its partially filled 3d orbitals, can promote electron or energy transfer from the excited CQDs, although its quenching efficiency is lower than that of Fe^2+^ under identical conditions.

**Fig. 5:**
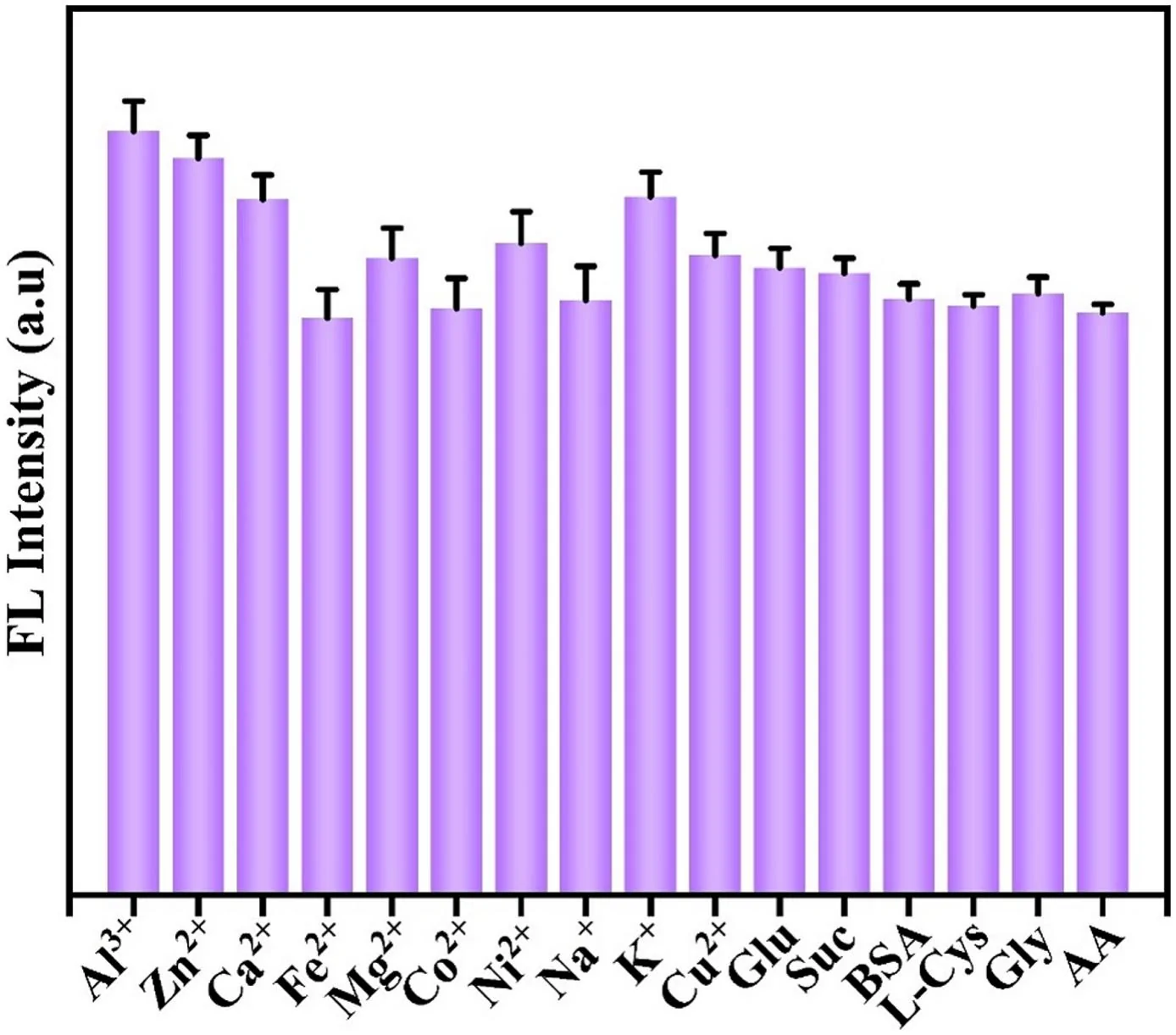
Effect of common interfering species on the fluorescence response of the BN-CQDs. The fluorescence intensity was recorded in the presence of various metal ions and biomolecules under optimized conditions. Data are shown as mean ± SD (n = 3).

The heteroatom-derived surface states improve charge distribution, reduce surface defects, and suppress nonspecific adsorption of foreign species, thereby preserving radiative excitation recombination. Boron, as an electron-deficient dopant, introduces acceptor states, whereas nitrogen contributes electron-rich donor states, together creating highly stable luminescent centers with reduced susceptibility to external perturbations. Consequently, weakly coordinating ions, including K^+^, Na^+^, Mg^2+^, and biomolecules, including glucose, sucrose, BSA, L-cysteine, glycine, and ascorbic acid, are unable to effectively compete for the surface-active sites for fluorescence emission. The negligible fluorescence variation observed in the presence of these interferents demonstrates the outstanding anti-interference performance of the synthesized BN-CQDs. Therefore, the synthesized BN-CQDs exhibit excellent resistance to nonspecific interference, making them highly promising fluorescent nanoprobes for sensing in complex environmental matrices.

### 3.7 Selectivity toward different antibiotics and interferents

The selectivity of the BN-CQDs toward different antibiotics, including AMO, CIP, TC, LVX, SMZ, OTC, SM, KAN, and interferents under identical experimental conditions (Fig. 6). The fluorescence intensity of the blank BN-CQDs remained the highest, whereas the addition of CIP and LVX resulted in a larger decrease in fluorescence emission, demonstrating its superior quenching efficiency compared to other antibiotics. In contrast, TC and AMO produced only moderate fluorescence attenuation, while OTC, SMZ, SM, and KAN caused negligible changes in fluorescence intensity. The distinct fluorescence response toward CIP and LVX confirms that the synthesized BN-CQDs possess excellent selectivity, enabling effective selection of CIP even in the presence of structurally related antibiotics. The remarkable selectivity of BN-CQDs toward CIP and LVX can be attributed to the synergistic contribution of heteroatom doping and favorable molecular interactions between CIP and surface-active sites of the CQDs. XPS and FTIR analyses confirmed the presence of abundant hydroxyl, carboxyl, amino, and boron-containing functional groups, which provide multiple interaction sites for CIP molecules. CIP and LVX possess a rigid quinolone aromatic framework together with carbonyl and carboxyl functionalities that facilitate stronger π-π interactions, hydrogen bonding, and electrostatic attraction with the surface of BN-CQDs. These interactions promote efficient PET and non-radiative charge transfer from the excited BN-CQDs to CIP and LVX, resulting in significant fluorescence quenching [43]. Furthermore, boron and nitrogen co-doping modify the electronic structures of the carbon framework by introducing electron-accepting and electron-donating surface states, thereby enhancing the sensitivity of emissive centers toward CIP and LVX while minimizing nonspecific interactions with other antibiotics [44]. Similar selective fluorescence quenching mechanisms have been reported for heteroatom-doped CQDs, where the combination of surface functional groups and tailored electronic properties governs the analyte recognition through selective charge-transfer pathways rather than simple adsorption. To further validate the robustness and specificity of the BN-CQDs, the influence of potentially competing ions and small molecules in the presence on the fluorescence response toward CIP and LVX was investigated (Fig. S7). The presence of the tested interferents produced only minor changes in the fluorescence quenching profiles of CIP and LVX. This result confirms that the BN-CQDs maintain a reliable response toward both fluoroquinolone antibiotics under competitive conditions.

**Fig. 6:**
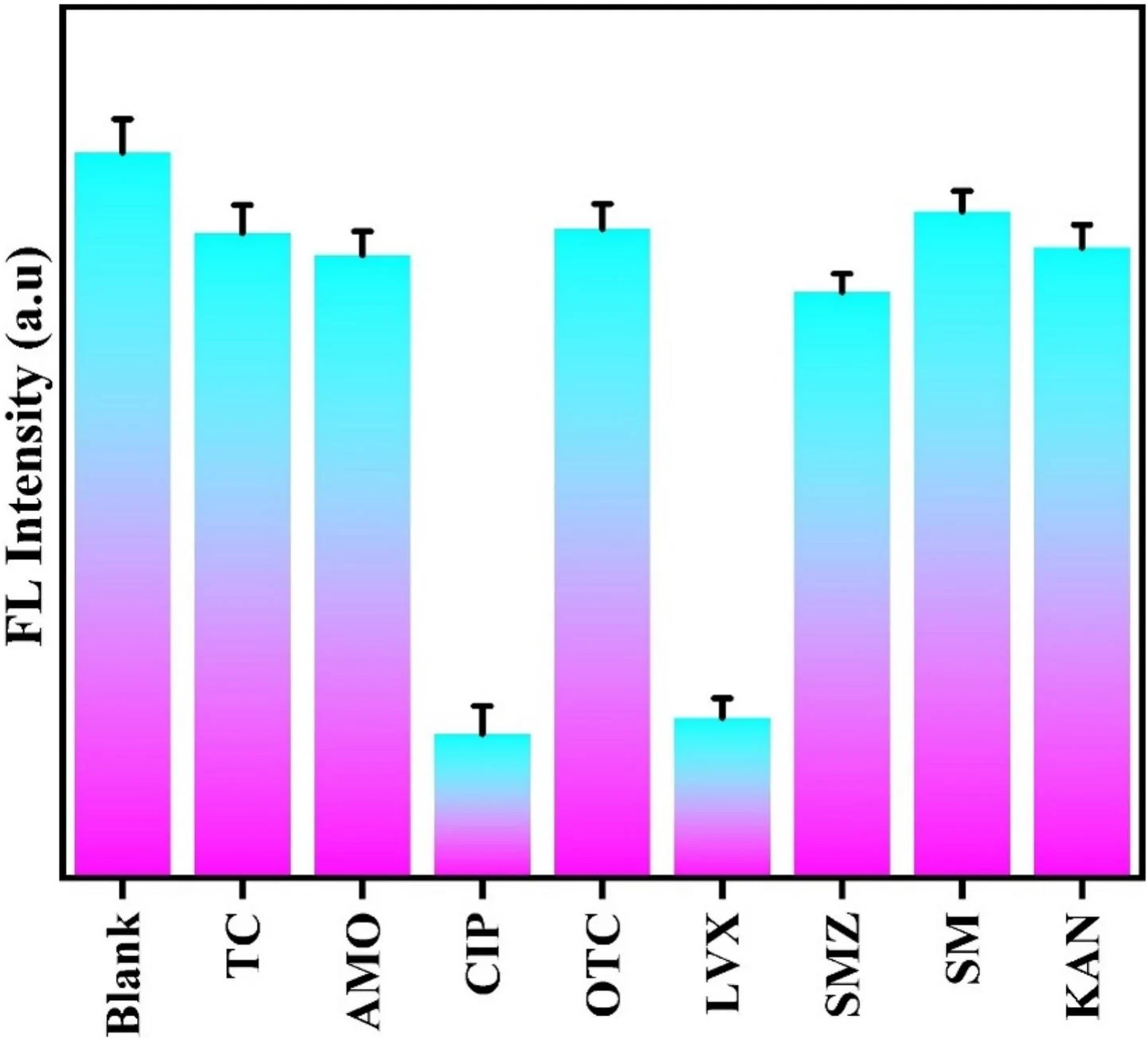
Selectivity of the BN-CQDs toward CIP and LVX in the presence of other antibiotics.

### 3.8 Fluorescence sensing of fluoroquinolone antibiotics

#### 3.8.1 Sensitivity toward CIP

The analytical sensitivity of the synthesized BN-CQDs toward CIP was evaluated by monitoring the fluorescence response over a concentration range of 0 to 50 μmol L^−1^ (Fig. 7a). As the concentration of CIP increased, the fluorescence intensity of the BN-CQDs decreased progressively without any noticeable shift in the emission maximum, indicating that the interaction between CIP and CQDs. This interaction does not alter the emissive surface states but instead promotes fluorescence quenching through non-radiative decay pathways. CIP-induced quenching of BN-CQDs photoluminescence exhibits concentration-dependent behaviour. The efficient quenching is attributed to the strong interaction between CIP molecules and abundant surface functional groups, including hydroxyl, carboxyl, amino, and boron-containing moieties, which facilitate the PET and charge transfer process. Similar sensing mechanisms have been reported for heteroatom-doped CQDs employed in antibiotic detection, where the synergistic effect of surface functionalization and heteroatom doping significantly improves sensing efficiency. The quantitative sensing performance was further evaluated using the Stern-Volmer relationship by plotting F_0_/F against the concentration of CIP (Fig. 7b). A good linear correlation was obtained within the concentration range of 0 to 50 μmol L^−1^, following the regression of F_0_/F = 0.105 + 1.090 with a correlation coefficient of R^2^ = 0.98, indicating excellent linearity and reproducibility of the sensing platform. The enlarged inset further demonstrates that the sensor exhibits a measurable fluorescence response even at low CIP concentrations, confirming its capability for trace-level detection. The limit of detection (LOD) was calculated based on the 3σ/k criterion. The LOD was 0.286 μmol L^−1^, demonstrating the high analytical sensitivity of the developed fluorescent probe. The low LOD can be attributed to the high fluorescence quantum yield of the BN-CQDs and the abundance of accessible surface active sites. The analytical performance compared with previously reported CQD-based platforms for antibiotics (Table S3), highlights the potential of the synthesized BN-CQDs as a rapid, sensitive, and reliable fluorescent sensing platform for monitoring CIP in environmental samples.

**Fig. 7:**
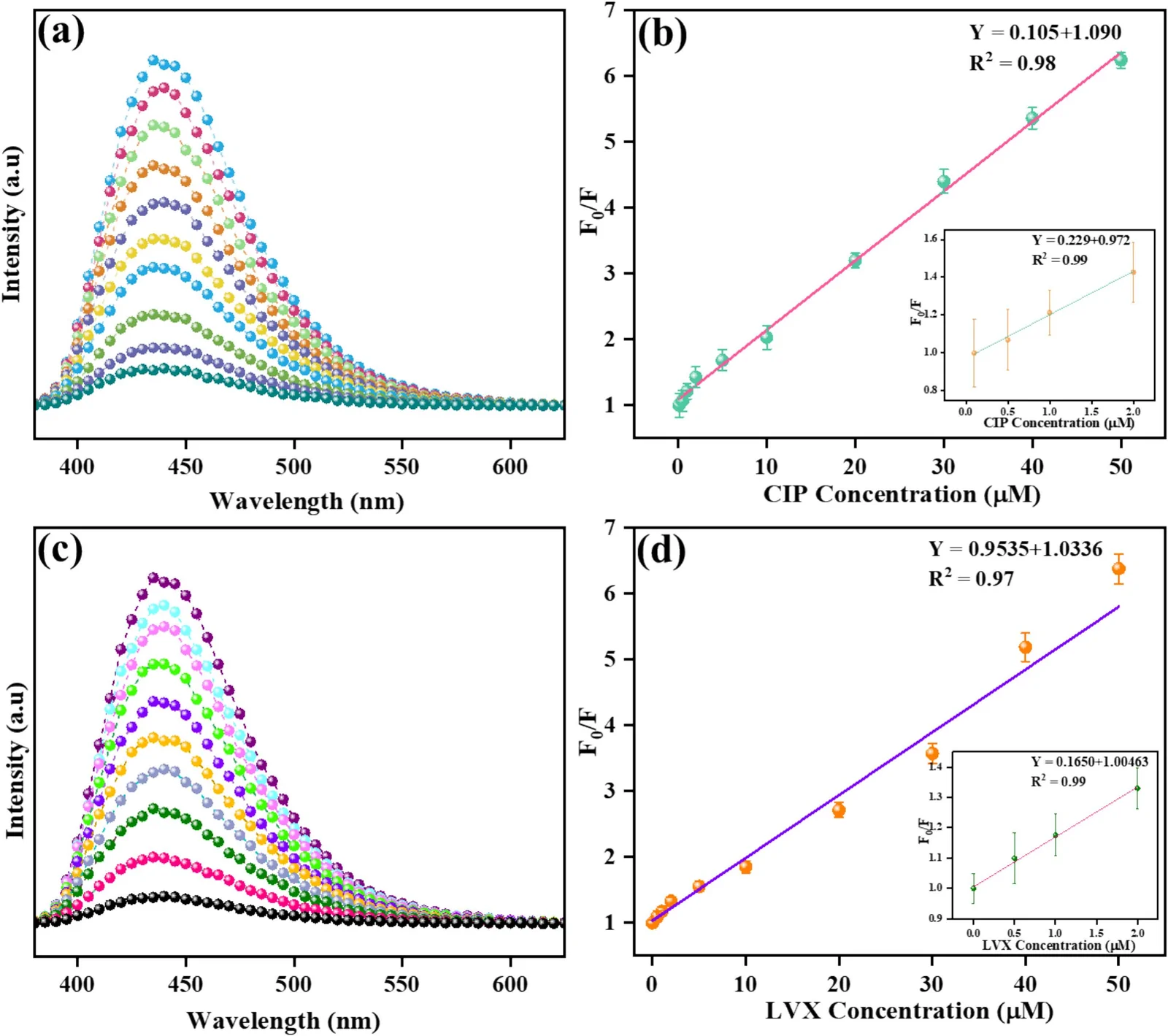
Fluorescence response of the BN-CQDs toward CIP detection. (a,c) Emission spectra in the presence of increasing concentrations of CIP and LVX. (b,d) Corresponding Stern-Volmer calibration curves obtained by plotting F_0_/F versus CIP and LVX concentrations, with the inset illustrating the linear relationship at low CIP and LVX concentrations.

The sensing performance of the BN-CQDs toward LVX was investigated under optimized experimental conditions by monitoring the concentration-dependent fluorescence response. The fluorescence intensity of the BN-CQDs decreased systematically after the gradual addition of LVX, demonstrating an efficient quenching process over the investigated concentration range. A good linear correlation was obtained within the concentration range of 0 to 50 μmol L^−1^, following the regression of F_0_/F = 0.9535 + 1.090 with a correlation coefficient of R^2^ = 0.97, indicating excellent linearity and reproducibility of the sensing platform. The LOD was determined to be 0.320 μmol L^−1^, demonstrating the high analytical sensitivity of the developed fluorescent probe [5]. The rapid fluorescence response indicates a strong affinity between LVX molecules and the surface of the BN-CQDs, enabling sensitive quantitative determination. The prominent response toward LVX suggests the formation of favorable interfacial interactions between the antibiotic and the heteroatom-enriched carbon nanostructure. This enhanced sensing performance can be attributed to the unique molecular structure of LVX, which contains a fluoroquinolone core together with a fused oxazine ring and multiple oxygen- and nitrogen- containing functional groups capable of interacting with the electron-rich surface of the BN-CQDs. The coexistence of electron-deficient boron sites and electron-rich nitrogen sites provides multiple adsorption centers that promote efficient excited-state electron transfer, resulting in concentration-dependent fluorescence quenching. Such cooperative interactions have been widely recognized as an important factor for the fluorescence response of heteroatom-doped CQDs toward fluoroquinolone antibiotics.

### 3.9 Sensing mechanism

To elucidate the fluorescence quenching mechanism of BN-CQDs toward fluoroquinolone antibiotics, the interaction between the fluorescent probe and the target analyte was evaluated based on their photophysical characteristics. The gradual decrease in fluorescence intensity with increasing CIP and LVX concentrations indicates an effective interaction between the antibiotics and the emissive surface states of BN-CQDs. The adsorption characteristics of the antibiotics may also contribute to the observed response through the inner filter effect (IFE), particularly when spectral overlap occurs between the excitation regions of BN-CQDs and the antibiotic absorption bands. Similar fluorescence-sensing studies of CQDs have shown that spectral overlap and fluorophore-analyte interactions can contribute to fluorescence quenching (Fig. 8) [3]. The heteroatom-rich surface of BN-CQDs provides potential interaction sites for CIP and LVX, promoting close association between the antibiotic molecules and the fluorescent surface states. Accordingly, the fluorescence quenching observed in the present investigation is proposed to arise from a combination of analyte-surface interactions, possible IFE, and electron transfer-mediated quenching, rather than being attributed exclusively to a single mechanism. The good linear Stern-Volmer relationships for CIP and LVX further confirm concentration-dependent fluorescence quenching. The associated electron transfer process may suppress the radiative recombination in excited BN-CQDs states, thereby promoting nonradiative relaxation and resulting in efficient fluorescence quenching [6]. The absence of an apparent shift in the emission wavelength further suggests that the emissive surface states of the BN-CQDs remain unchanged during sensing.

**Fig. 8:**
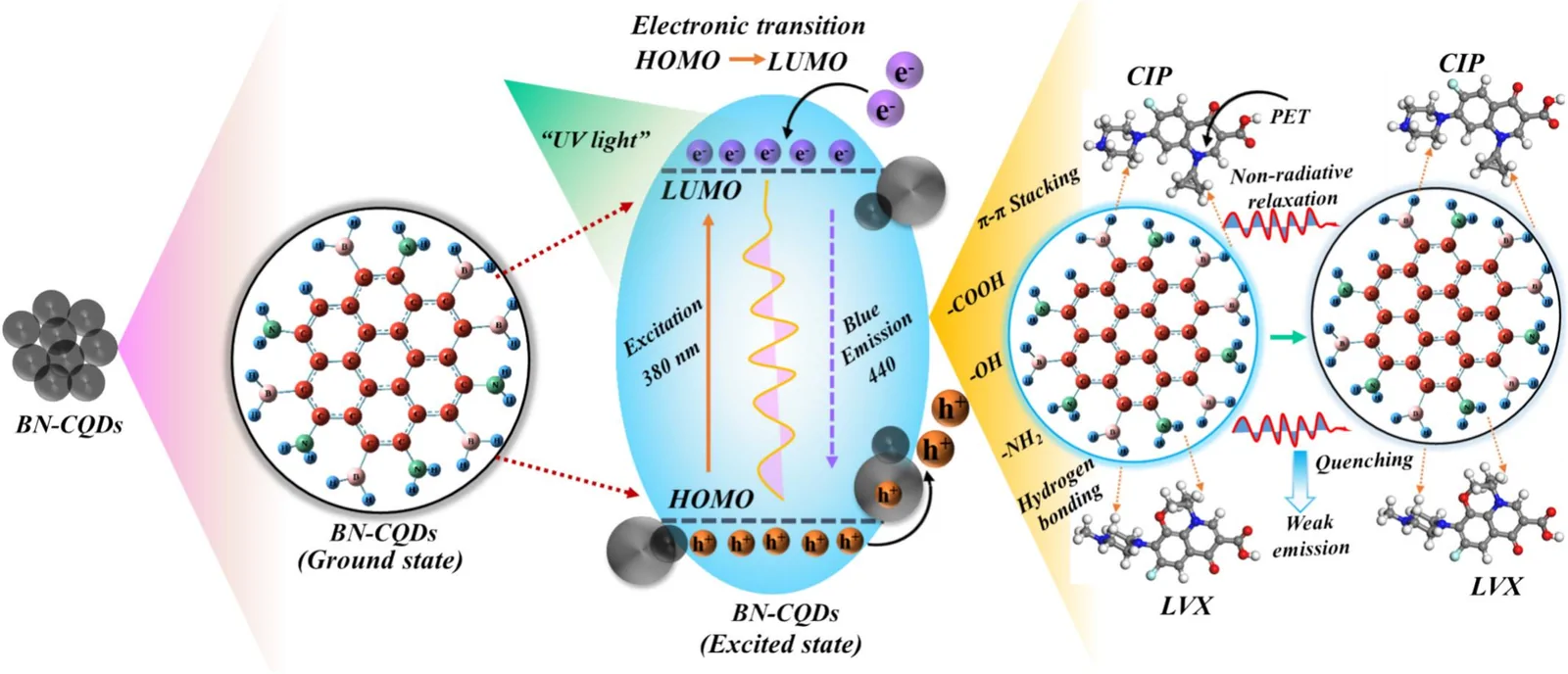
Proposed fluorescence sensing mechanism of BN-CQDs toward fluoroquinolone antibiotics.

### 3.10 DFT-guided mechanistic insights

To gain further insights into the electronic structure and sensing mechanism of the BN-CQDs toward fluoroquinolone antibiotics, DFT calculations were performed to investigate the electronic structures, frontier molecular orbitals, and electrostatic potential distributions of BN-CQDs, CIP, and LVX. The optimized molecular geometries of CIP and LVX, frontier molecular orbitals (HOMO and LUMO), and ESP potential surfaces were calculated using *Gaussian 09* and visualized with *GaussView 6.0*. These theoretical analyses provide valuable insights into the molecular interactions governing the fluorescence sensing process. The optimized molecular structures of CIP and LVX fluoroquinolone backbones feature multiple oxygen- and nitrogen-containing functional groups capable of interacting with the surface functionalities of BN-CQDs through hydrogen bonding and electrostatic attraction. Although both antibiotics share a similar molecular framework, the presence of a fused oxazine ring in LVX slightly modifies its molecular geometry and electronic distribution, which is expected to influence its interaction with the fluorescent probe.

The changes in the electronic states of the molecules provide valuable insights into the analyte interactions and underlying sensing mechanism. Therefore, to investigate the electronic changes induced by the adsorption of CIP and LVX on BN-CQDs, the HOMO and LUMO distributions of the isolated BN-CQDs, CIP, and LVX molecules and their corresponding BN-CQD/CIP and BN-CQD/LVX complexes, were analyzed and compared before and after complex formation. This analysis provides insight into the redistribution of frontier molecular orbitals and the electronic interactions occurring upon analyte binding. Frontier molecular orbital analysis further illustrates that the HOMO and LUMO are mainly distributed over the conjugated quinolone framework and the heterocyclic nitrogen-containing moieties of both antibiotics. For the pristine BN-CQDs, the HOMO and LUMO energies were calculated to be about −5.11 and −1.74 eV, respectively, corresponding to a HOMO-LUMO gap of 3.37 eV (Fig. S8a–d). CIP exhibited HOMO and LUMO energies of −5.03 eV and −1.76 eV, respectively, with a corresponding energy gap of 3.27 eV (Fig. S8e–h), whereas LVX showed HOMO and LUMO energies of −5.71 eV and 2.01 eV, respectively, resulting in an energy gap of 3.70 eV (Fig. S8i–l). These results establish the distinct electronic characteristics of the individual sensing components before complex formation. Upon complex formation, noticeable changes in frontier orbital energies and distributions were observed. For the optimized BN-CQDs/CIP complex, the HOMO and LUMO energies shifted to −5.13 and −2.01 eV, respectively, reducing the HOMO-LUMO gap to 3.12 eV (Fig. 9a–d). Similarly, the BN-CQDs/LVX complex exhibited HOMO and LUMO energies of −5.22 and −2.16 eV, respectively, with a reduced energy gap of 3.06 eV (Fig. 9e–h). The reduction in the HOMO-LUMO gap from 3.38 eV (BN-CQDs) to 3.12 and 3.06 eV following complex formation with CIP and LVX indicates electronic perturbations and enhanced electronic coupling upon antibiotic binding.

**Fig. 9:**
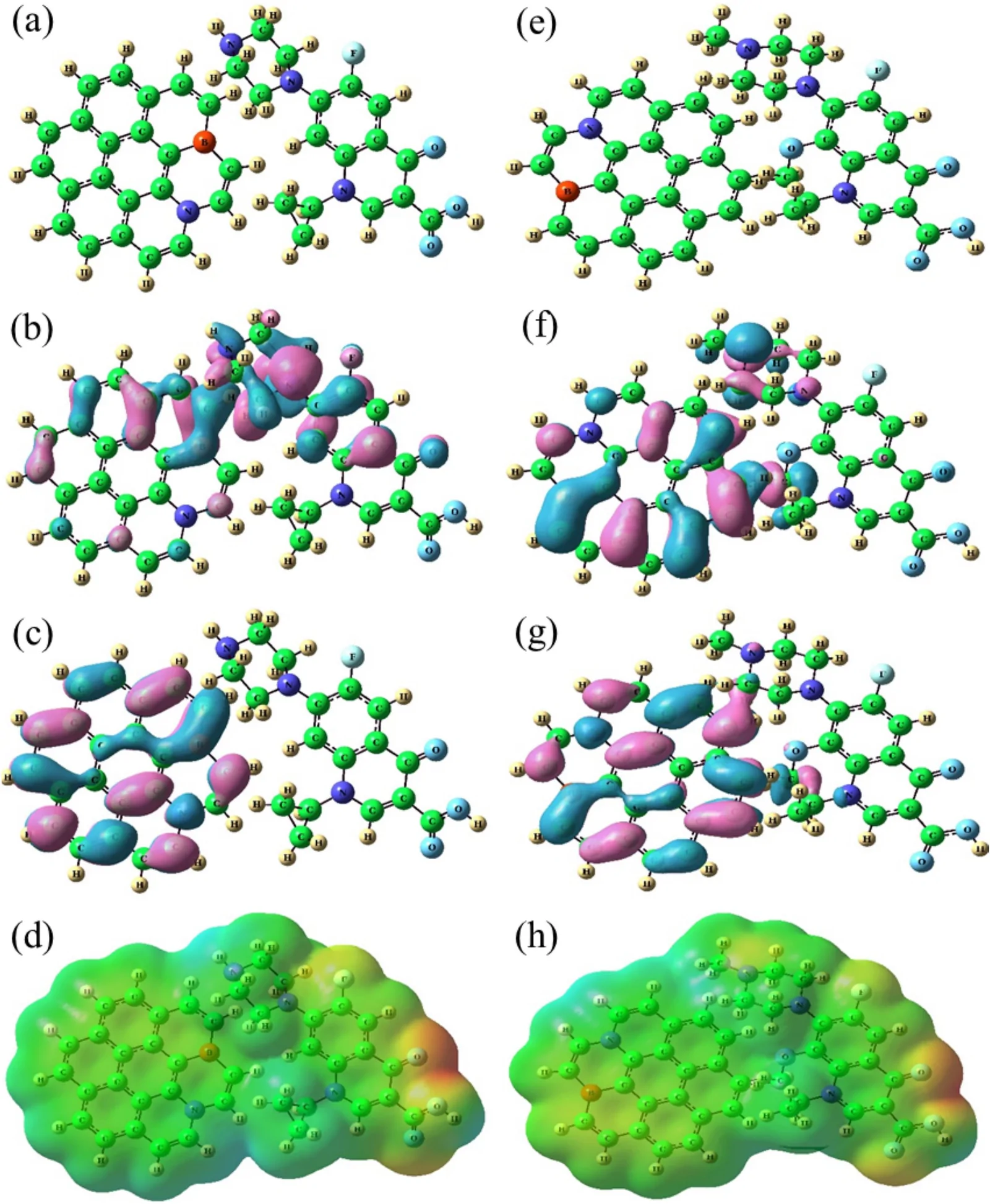
DFT analysis of the optimized BN-CQDs/CIP and BN-CQDs/LVX complexes. (a, e) Optimized structures of the BN-CQDs/CIP and BN-CQDs/LVX complexes. (b,f) HOMO distributions. (c,g) LUMO distributions. (d,h) Molecular electrostatic potential (MEP) maps. The calculated frontier molecular orbital distributions and MEP maps provide insights into the electronic structure, charge distribution, and possible intermolecular interactions of the CQDs with CIP and LVX.

The altered frontier molecular distributions in the complexes suggest that the absorbed antibiotic molecules, supporting effective interfacial electronic interaction, influence the electronic states of the BN-CQD framework. Upon excitation of the BN-CQDs at 380 nm, electrons are excited and transferred to the electron-accepting molecular orbitals of the adsorbed antibiotic molecules. The photoinduced electron transfer may suppress the radiative recombination of excited charge carriers, resulting in efficient fluorescence quenching. The calculated frontier orbital distributions therefore provide theoretical support for the experimentally observed concentration-dependent fluorescence intensity. ESP potential analysis further elucidates the adsorption behavior of the antibiotics on the BN-CQDs surface (Fig. 9d and h). The negative electrostatic potential is predominantly localized around the carbonyl, oxygen, carboxyl oxygen, and fluorine-containing regions, whereas the positive electrostatic potential is mainly positioned around the protonated nitrogen atoms of the piperazine and heterocyclic rings. This complementary charge distribution promotes strong electrostatic interactions with the hydroxyl, amino, carboxyl, and boron-containing functional groups present on the BN-CQDs. These interactions facilitate the formation of a stable ground-state association between the fluorescent probe and the analyte, bringing the molecules into proximity and thereby enhancing the efficiency of electron transfer. The combined experimental and theoretical results confirm that the adsorption of CIP and LVX onto the BN-CQDs surface promotes efficient photoinduced electron transfer is the dominant pathway responsible for fluorescence quenching. The combination of heteroatom-induced surface active sites and favorable electronic interactions enables the synthesized BN-CQDs to serve as an efficient fluorescent platform for the sensitive detection of fluoroquinolone antibiotics.

### 3.11 Real sample analysis

#### 3.11.1 Sensing of CIP

To evaluate the practical applicability of the synthesized BN-CQDs, real-sample analysis was performed using drinking water and tap water samples spiked with different concentrations of CIP (0, 0.5, 5, 10, and 20 μmol L^−1^). Before analysis, the water samples were filtered to remove suspended impurities and then spiked with known amounts of CIP. The fluorescence response of the BN-CQDs was subsequently measured under the optimized experimental conditions, and the CIP concentration was determined from the corresponding calibration curve. As summarized in Table 1, the recoveries for drinking water ranged from 98.0 to 101.20%, while those for tap water ranged from 99.2 to 102.0%. These recoveries were close to 100%, indicating excellent analytical accuracy and negligible matrix interference during the determination of CIP in real water samples.

**Table 1:** Recovery analysis of spiked CIP in real samples.

Sample	CIP added (μmol L^−1^)	CIP detected (μmol L^−1^)	Recovery (%)	RSD (%)
Drinking water	0	N.D*	−	−
	0.5	0.49	98 0.0	2.15
	5	5.06	101.20	1.46
	10	9.86	98.60	1.21
	20	20.16	100.80	0.98
Tap water	0	N.D*	−	−
	0.5	0.51	102.0	2.08
	5	4.96	99.20	1.54
	10	10.06	100.60	1.13
	20	19.89	99.45	0.92

* Not detected.

#### 3.11.2 Sensing of LVX

To evaluate the practical applicability of the BN-CQDs, the proposed sensing platform was employed for the detection of LVX in real water samples, including drinking and tap water. The samples were analyzed using the standard addition method by spiking known concentrations of LVX, and the analytical results were summarized in Table 2. No detectable LVX signal was observed in the unspiked water samples, indicating the absence of background interference and confirming that the selected water matrices were free from detectable levels of LVX. The recoveries obtained for water samples ranged from 96.0% to 102.6% for all measurements. The satisfactory recovery values demonstrate the excellent accuracy of the proposed sensing method, while the Low RSD values indicate good precision and reproducibility. The slight variations in recovery are within the acceptable range for fluorescence-based analytical methods and can be attributed to minor matrix effects commonly encountered in environmental water samples. Importantly, these matrix components did not significantly influence the fluorescence response of the BN-CQDs, highlighting the robustness and selectivity of the sensing platform.

**Table 2:** Recovery analysis of spiked LVX in real samples.

Sample	LXV added (μmol L^−1^)	LXV detected (μmol L^−1^)	Recovery (%)	RSD (%)
Drinking water	0	N.D	−	−
	0.5	0.48	96.0	2.84
	5	4.92	98.40	2.31
	10	10.12	101.20	2.08
	20	19.92	99.60	1.96
Tap water	0	N.D	−	−
	0.5	0.51	102.0	2.76
	5	5.13	102.60	2.18
	10	9.87	98.70	2.05
	20	20.24	101.20	1.89

The satisfactory recoveries demonstrate that the complex chemical constituents naturally present in drinking and tap water did not significantly affect the fluorescence response of the BN-CQDs. This excellent analytical performance can be attributed to the abundant oxygen-, nitrogen-, and boron-containing surface functional groups, which provide selective interaction sites for fluoroquinolones while minimizing nonspecific adsorption of other dissolved species. In addition, the heteroatom-induced modification of the electronic structure enhances the stability of the emissive surface states, resulting in a robust fluorescence response even in complex sample matrices [6], [8], [45]. The low RSD values obtained from repeated measurements further confirm the excellent precision and reproducibility of the sensing platform. These high recovery values, satisfactory precision, and minimal matrix effects demonstrate that the developed BN-CQDs are a reliable and sensitive fluorescent probe for the quantitative determination of fluoroquinolones in water samples.

## 4 Conclusion

The highly fluorescent BN-CQDs sensing probe was successfully prepared using an ultrasonic-assisted hydrothermal approach. US irradiation influenced the physicochemical properties of the synthesized BN-CQDs, as evidenced by the observed changes in their optical, structural, and morphological characteristics. The synthesized BN-CQDs exhibited excellent optical characteristics, with a fluorescence quantum yield of 56.8%, highlighting the beneficial role of B and N co-doping in improving the photoluminescence properties of CQDs. Comprehensive surface and structural characterization confirmed the incorporation of heteroatoms and the presence of abundant oxygen- and nitrogen-containing functional groups, which provide active sites for efficient interaction with CIP and LVX. The resulting BN-CQDs enabled fluorescence-based sensing of both CIP and LVX over a range of 0 to 50 μmol L^−1^, with detection limits of 0.286 μmol L^−1^ and 0.320 μmol L^−1^. The sensing platform exhibited excellent selectivity against structurally related antibiotics, metal ions, and biomolecules. The combined experimental and theoretical results confirm that the adsorption of CIP and LVX onto the BN-CQDs surface promotes efficient photoinduced electron transfer is the dominant pathway responsible for fluorescence quenching. The satisfactory recoveries obtained from the real water samples demonstrate the analytical reliability of the present method. Compared with previously reported CQDs sensing probes, the present study offers a dual-fluoroquinolone sensing platform integrating ultrasonic-assisted synthesis and high photoluminescence efficiency. The combination of a simple synthesis process, tailored optical performance, high selectivity, and reliable applicability provides a practical foundation for fluorescence-based monitoring of antibiotics in water systems and offers opportunities for the future development of portable and field-deployable sensing systems.

## CRediT authorship contribution statement

**Duraisamy Elango:** Writing – review & editing, Writing – original draft, Data curation, Conceptualization. **Velu Subash:** Investigation, Formal analysis, Data curation. **Palaniyappan Jayanthi:** Validation, Investigation, Formal analysis. **Velu Manikandan:** Writing – review & editing, Validation, Formal analysis, Data curation. **Kwang Soup Song:** Writing – review & editing, Validation, Supervision, Funding acquisition.

## Declaration of competing interest

The authors declare that they have no known competing financial interests or personal relationships that could have appeared to influence the work reported in this paper.

## Data Availability

The data that support the findings of this study are available from the corresponding author upon reasonable request.

## Appendix A Supplementary data

Supplementary Data 1 Appendix A provides supplementary data, including additional experimental results, characterization data, figures, tables, and supporting information relevant to the findings of this study.
