# Supplementary material for: Ultrasound-assisted synthesis of surface state engineered BN-carbon quantum dots for fluoroquinolone sensing: DFT insights
Source: Ultrason Sonochem. 2026 Oct 2;134:108094. doi: 10.1016/j.ultsonch.2026.108094 (PMC13671363; doi:10.1016/j.ultsonch.2026.108094)
Supplement: Supplementary file 1 [file mmc1.docx]

**Supporting Information**

**Methodology**

***2.5. Quantum yield measurement***

The photoluminescence quantum yield (QY) of synthesized BN-CQDS was evaluated by a comparative approach using quinine sulfate dissolved in 0.1 M sulfuric acid as the standard fluorophore (Φ = 0.54). All optical measurements of both the sample and reference were performed under identical instrumental conditions to ensure accuracy and comparability. Prior to measurement, a series of both BN-CQDS and quinine sulfate was prepared to obtain absorbance values below 0.05. This low-absorbance condition was maintained to suppress inner filter effects and eliminate deviations due to self-absorption. The quantum yield of BN-CQDS was calculated based on the proportional relationship between fluorescence emission and optical absorption using the standard comparative expression (Equation S1). $QY_{BN-CQDS}=QY_{R}\frac{I_{N}}{I_{R}}\frac{A_{R}}{A_{N}}\left(\frac{\eta_{N}}{\eta_{R}}\right)^{2}$ (S1)

Where *QY* is the fluorescence quantum yield; I is the integrated emission spectral area; A is the absorbance under incident light at this excitation wavelength; η is the refractive index of the solution; subscript N denotes BN-CQDS; and subscript R denotes the reference material.

***2.6. Optimization of sensing conditions and stability of BN-CQDs***

The analytical conditions were systematically optimized by investigating the effects of pH, incubation time, and probe concentration. The pH-dependent fluorescence response was evaluated under different pH conditions (6-10), while the effects of incubation time (0-30 min) and BN-CQDs concentration (0.05-0.3 mg mL^-1^) were investigated under identical experimental conditions. The fluorescence response was recorded for each condition, and optimum parameters were selected based on the magnitude and stability of the fluorescence quenching response. The optimized conditions were subsequently employed for the fluorescence sensing experiments. The stability of the synthesized BN-CQDS was comprehensively evaluated by investigating its fluorescence performance under different storage conditions, ultraviolet (UV) irradiation, solution pH, and ionic strength. All fluorescence measurements were carried out under identical instrumental conditions using the optimized excitation wavelength, and the relative fluorescence intensity was used to evaluate the stability of the BN-CQDS. To assess the storage stability, the purified BN-CQDS dispersion was stored in sealed amber glass vials at room temperature for 30 days. Samples were withdrawn at regular 2-day intervals, and their fluorescence emission spectra were recorded. The fluorescence intensity retained after storage was compared with that of freshly prepared samples to evaluate the long-term stability of the CQDs. The photostability of BN-CQDS was investigated by continuously exposing the CQDs solution to a 365 nm UV lamp at room temperature for 12 hrs. Fluorescence spectra were recorded at 2-hour intervals to assess the fluorescent probe's photobleaching resistance during prolonged UV exposure. The effect of the fluorescence behavior of the BN-CQDS was examined over a pH range of 6.0-10.0. The pH of the solutions was adjusted using 0.1 N HCl and 0.1 M NaOH. After mixing the CQDs with the corresponding buffer and equilibrating for 15 min at room temperature, the fluorescence emission spectra were recorded to evaluate the influence of acidic and alkaline environments on fluorescence stability. The tolerance of the BN-CQDS toward variations in ionic strength was evaluated using NaCl solutions. Equal volumes of CQDs solution and NaCl solutions of different concentrations were mixed and incubated for 15 min, and the fluorescence of the solutions was recorded. The resulting fluorescence intensities were compared with the control sample to determine the effect of electrolyte concentration on the optical stability of the BN-CQDS. All experiments were performed in triplicate, and the results are reported as the mean ± standard deviation, demonstrating the stability and robustness of the synthesized BN-CQDS under various environmental and storage conditions.

**2.7. Anti-interference study**

The interference resistance of the prepared BN-CQDS was investigated by examining their fluorescence response in the presence of commonly occurring inorganic ions and biological molecules that may coexist in environmental and biological samples. The objective was to evaluate whether these substances could influence the fluorescence behavior of the BN-CQDS under the optimized sensing conditions. Stock solutions (1 mM) of the metal ions K^+^, Na^+^, Ca^2+^, Mg^2+^, Al^3+^, Fe^2+^, Co^2+^, Ni^2+^, Cu^2+^, and Zn^2+^ were prepared by dissolving the corresponding analytical-grade salts in ultrapure water. Similarly, solutions of representative biomolecules, including glucose, sucrose, BSA, L-cysteine, glycine, and ascorbic acid, were freshly prepared at the same concentration. For each experiment, an appropriate volume of the BN-CQDS solution was mixed with 10 mM PBS. We then added each interfering species separately to reach a final concentration of 100 μM. This mixture was gently vortexed and incubated for 10 min at room temperature to ensure sufficient interaction between the interfering species and the surface functional groups of the BN-CQDS. Following incubation, the fluorescence emission spectra were recorded using the optimized excitation wavelength, and the fluorescence intensity at the maximum emission wavelength was measured. Each experiment was performed in triplicate, and the results are reported as the mean ± standard deviation, demonstrating the robustness of the BN-CQDS toward applicability for analysis in complex sample matrices.

***2.8. Selectivity toward different antibiotics and interferents***

The selectivity of synthesized BN-CQDS toward Fluoroquinolones (CIP and LVX) was investigated by comparing its fluorescence response with that of several commonly detected antibiotics and interferents under identical experimental conditions. AMO, TC, SMZ, OTC, SM, and KAN were selected as potential interfering antibiotics because they are frequently encountered in environmental and pharmaceutical samples and possess different molecular and functional groups. Stock solutions (1.0 mM) of each antibiotic were prepared using ultrapure water and stored at 4 ºC until use. For the selectivity assay, 100 μL of BN-CQDS (0.1 mg mL^-1^) was transferred to an amber glass vial, followed by the addition of equal volumes of each antibiotic (10 μM), and the total reaction volume was adjusted to 3.0 mL with PBS (10 mM) to maintain identical experimental conditions for all analytes. These mixtures were gently vortexed and incubated at room temperature for 15 min to ensure sufficient interaction between the antibiotics and fluorescent probe. The fluorescence emission spectra were subsequently recorded using a fluorescence spectrophotometer at the optimized excitation wavelength, and the emission intensity was monitored. For an anti-interference study, 100 μL of BN-CQDS together with CIP or LVX and selected potentially interfering metal ions and small molecules were added to the reaction mixture. The fluorescence responses were compared with those of CIP and LVX in the absence of interferents to evaluate the anti-interference capability of BN-CQDs.

***2.9. Sensitivity toward CIP and LVX detection***

The sensitivity of CIP and LVX on the fluorescence response of the BN-CQDS sensing platform was systematically investigated under optimized experimental conditions. A stock solution of CIP and LVX was prepared in ultrapure water and subsequently diluted to obtain a series of working solutions with concentrations ranging from 0-50 μM. For each measurement, 100 μL of BN-CQDS (0.1 mg mL^-1^) was transferred to an ambered glass vial followed by the addition of various concentrations of CIP and LVX**;** the total reaction volume was adjusted to 3.0 mL using PBS (10 mM) to ensure identical experimental conditions for all measurements. The mixtures were gently vortexed and incubated at room temperature for 15 min to ensure sufficient interaction between the fluorescent probe and CIP and LVX molecules. The fluorescence emission spectra were subsequently recorded using a fluorescence spectrophotometer at the optimized excitation wavelength, and the emission intensity was monitored.

***2.10. Density functional theory (DFT) calculations***

To elucidate molecular-level insight into the fluorescence sensing behaviour of BN-CQDS, CIP and LVX, DFT calculations were performed to investigate their electronic structures, frontier molecular orbitals (FMO), and electrostatic potential distributions. The theoretical calculations were performed using the *Gaussian* *09* computational package, and the optimized molecular structures together with the electronic properties were visualized using *GaussView 6.0*. DFT analysis was employed to establish the relationship between the electronic characterization of the antibiotic molecules and their fluorescence quenching behaviour toward the synthesized BN-CQDs. The initial molecular geometries of CIP and LVX were constructed based on their reported crystal structures and subjected to full geometry optimization without any geometrical constraints. Geometry optimization was performed using the hybrid Becke-three-parameter Lee-Yang-Parr (B3LYP) exchange-correlation functional together with the 6-31G(d,p) basis set, which has been extensively employed for the electronic structure analysis of organic molecules containing carbon, oxygen, nitrogen, and fluorine atoms. After structural optimization, FMO analysis was performed to determine the energies and spatial distributions of the highest occupied molecular orbital (HOMO) and the lowest unoccupied molecular orbital (LUMO). The HOMO energy represents the electron-donating capability of the molecule, whereas the LUMO energy reflects the electron-accepting tendency. The HOMO-LUMO energy gap (ΔE) was subsequently calculated to evaluate the electronic excitation characteristics and chemical reactivity of the investigated antibiotics. Since fluorescence quenching generally involves intermolecular charge transfer between the fluorescent probe and the target analyte, the frontier orbital energies provide valuable information in the sensing process. To further understand the interaction between the antibiotic molecules and the surface functional groups of the BN-CQDs, molecular electrostatic potential (ESP) surfaces were generated based on the optimized electron density. The ESP maps were employed to visualize the distribution of positive and negative ESP potential over the molecular surface, thereby identifying electron-rich and electron-deficient regions that are favorable for intermolecular interactions. These DFT calculations complement the experimental observations and provide a theoretical basis for the high sensitivity and selectivity of the BN-CQDs toward fluoroquinolone detection.

***2.11. Real sample analysis***

To evaluate the practical applicability of the developed BN-CQDs fluorescence sensing platform, the method was validated using real environmental water samples. Representative samples. Tap water and drinking water were collected in pre-cleaned polyethylene bottles. The samples were transported to the laboratory and stored at 4 ºC before analysis. Before analysis, all water samples were filtered through a 0.22 μM membrane filter to remove suspended particles and other insoluble impurities. Known concentrations of CIP were spiked into the filtered water samples at four concentration levels. Subsequently, 100 μL of BN-CQDs (0.1 mg mL^-1^) was mixed with an appropriate volume of each spiked sample, and the final volume was adjusted to 3.0 mL using PBS. The mixtures were gently vortexed and incubated at room temperature for 15 min to complete interaction between the BN-CQDs and the analyte. Following incubation, the fluorescence emission spectra were recorded using a fluorescence spectrophotometer at the optimized excitation wavelength, and the emission intensity was monitored. The CIP concentration of each sample was calculated from the previously established calibration curve. Each real sample was analysed in triplicate (n=3), and the mean value with the standard deviation was reported. The analytical performance of the method was evaluated by determining the percentage recovery and relative standard deviation (RSD %).

**Fig. S1.** Calorimetric measurement of the ultrasound system.

**Table - S1.** Calorimetric measurements of dissipated ultrasonic power and power density of the ultrasound system (mean ± SD, n=3)

| **Parameter** | **Value** |
| --- | --- |
| Dissipated power (W) | 2.27 ± 0.5 |
| Power density (W dm^-3^) | 75.7 ± 1.7 |

**Fig. S2.** Excitation-dependent fluorescence behaviour of BN-CQDs.

**Fig. S3.** Comparative characteristics of BN-CQDs prepared with and without US pretreatment. (a) UV-Vis absorption spectra, (b) Excitation and emission spectra of BN-CQDs-NS, (C) Excitation and emission spectra of BN-CQDs-US, and (d) XRD patterns of BN-CQDs-US and BN-CQDs-NS.

**Fig. S4.** TEM micrographs and particle-size distribution of BN-CQDs prepared with and without US pretreatment. (a,b) TEM images and (c) particle size distribution of BN-CQDs-NS. (d,e) TEM images and (f) particle size distribution of BN-CQDs-US.

**Fig. S5.** XPS survey spectrum of the synthesized BN-CQDs, confirming the presence of carbon (C), nitrogen (N), boron (B), and oxygen (O) as the major constituent elements.

**Fig. S6.** Optimization of the sensing conditions. (a) effect of pH, (b) effect of incubation time, and (c) effect of BN-CQDs concentration.

**Fig. S7.** Anti-interferent assessment of the BN-CQDs for the selective determination of (a) CIP and (b) LVX in the presence of potentially competing metal ions and small molecules.

**Fig. S8.** Illustration of DFT-optimized structures, frontier molecular orbitals (HOMO-LUMO), and ESP potential maps of (a-d) BN-CQDs, (e-h) CIP, and (i-l) LVX.

**Table - S2.** Comparison of optical properties and fluorescence quantum yields of heteroatom-doped CQDs

| **Material** | **Precursor** | **Synthesis method** | **Ex/Em (nm)** | **QY (%)** | **Reference** |
| --- | --- | --- | --- | --- | --- |
| FN CQDs | Citric acid, urea, and triethylamine hydrogen fluoride | Hydrothermal | 365/525 | 26.7 | [1] |
| NS-CQDs | Thiomalic acid and diethylenetriamine | Hydrothermal | 360/442 | 17.59 | [2] |
| N-CQDs | Citric acid and ethylenediamine with diaminocyclohexane | Hydrothermal | 360/460 | 73.10 | [3] |
| N-CQDs | Citric acid, diethylenetriamine | Hydrothermal | 350/439 | 62.99 | [4] |
| N-CQDs | Nitronaphthalene and ammonia | Hydrothermal | 390/447 | 13.72 | [5] |
| N-CQDs | Citric acid and 2-aminophenol | Hydrothermal | 350/412 | 89.7 | [6] |
| BN-CQDs | Citric acid, boric acid and L-serine | Microwave | 340.7/427.4 | 24 | [7] |
| BN-CQDs | Phenylenediamine and boric acid | Microwave | 409/564 | 13.79 | [8] |
| BN-CQDs-NS | Citric acid, boric acid, and urea | Hydrothermal | 378/439 | 47.96 | **This work** |
| BN-CQDs-US | Citric acid, boric acid, and urea | US-assisted hydrothermal | 380/440 | 56.8 | **This work** |

**Table - S3.** Comparison of heteroatom-doped CQDs for the detection of various antibiotics

| **Material** | **Synthesis method** | **Antibiotics** | **Linear Range** | **LOD** | **Reference** |
| --- | --- | --- | --- | --- | --- |
| BN-CQDs | Hydrothermal | Penicillin | 0-350 μM | 10 μM | [9] |
| SN-CQDs | Hydrothermal | Tetracycline | 1.88 - 60 μM | 0.56 μM | [10] |
| N-CQDs | Hydrothermal | Oxytetracycline | 0-30 μM | 0.265 μM | [11] |
| F-CQDs | Hydrothermal | Ciprofloxacin | 1-100 μM | 6.06 μM | [12] |
| B-CQDs | Hydrothermal | Amoxicillin | 1.43-429.12 μM | 0.825 μM | [13] |
| BN-CQDs | US-assisted hydrothermal | Ciprofloxacin | 0-50 μM | 0.286 μM | **This work** |
|  |  | Levofloxacin | 0-50 μM | 0.320 μM | **This work** |

**Reference**

[1] R. Li, Y. Yang, C. Li, J. Liao, S. Zhu, R. Liu, H. Zhu, Synthesis of starch-based F/N-doped carbon dots for enhanced detection of latent fingerprint tertiary structures, New J. Chem. 49 (2025), pp. 9096–9101

[2] S. Chandra, A.R. Chowdhuri, T.K. Mahto, D. Laha, S.K. Sahu, Sulphur and nitrogen doped carbon dots: A facile synthetic strategy for multicolour bioimaging, tiopronin sensing, and Hg²⁺ ion detection, Nano-Struct. Nano-Objects 12 (2017), pp. 10–18.

[3] O.M.K. Koç, A.E. Üzer, R.A. Apak, High quantum yield nitrogen-doped carbon quantum dot-based fluorescent probes for selective sensing of 2,4,6-trinitrotoluene, ACS Appl. Nano Mater. 5 (2022), Article 5868–5881.

[4] T. Liu, Y. Yan, Z. Chen, C. Zhao, N-Doped CQDs as a fluorescence probe for tiopronin detection in pharmaceutical formulations via static quenching, J. Fluoresc. (2026) 1–19.

[5] H. He, E. Shuang, T. Wen, J. Yao, X. Wang, C. He, Y. Yu, Employing novel N-doped graphene quantum dots to improve chloride binding of cement, Constr. Build. Mater. 401 (2023), Article 132944.

[6] D. Dagar, B.P. Kafle, A. Ladan, V. Shanmuganathan, H. Teng, Y.-L. Lee, Selective recognition of ciprofloxacin by carbon quantum dots and their underlying mechanism, Chem. Eng. J. (2026), Article 174227.

[7] P. Keerthana, A.R. Cherian, U. Sirimahachai, D.A. Thadathil, A. Varghese, G. Hegde, Detection of picric acid in industrial effluents using multifunctional green fluorescent B/N-carbon quantum dots, J. Environ. Chem. Eng. 10 (2022), Article 107209.

[8] Y. Wei, L. Chen, J. Wang, X. Liu, Y. Yang, S. Yu, Rapid synthesis of BN co-doped yellow emissive carbon quantum dots for cellular imaging, Opt. Mater. 100 (2020), Article 109647.

[9] M.A. Mousa, H.H. Abdelrahman, M.A. Fahmy, D.G. Ebrahim, A.H. Moustafa, Pure and doped carbon quantum dots as fluorescent probes for the detection of phenol compounds and antibiotics in aquariums, Sci. Rep. 13 (2023), Article 12863.

[10] Y. Fan, W. Qiao, W. Long, H. Chen, H. Fu, C. Zhou, Y. She, Detection of tetracycline antibiotics using fluorescent “turn-off” sensor based on S, N-doped carbon quantum dots, Spectrochim. Acta A Mol. Biomol. Spectrosc. 274 (2022), Article 121033.

[11] C. Wang, Q. Sun, M. Yang, E. Liu, W. Xue, J. Fan, Preparation of highly luminescent nitrogen-doped carbon quantum dots and their detection of tetracycline antibiotics, Colloids Surf. A Physicochem. Eng. Asp. 653 (2022), Article 129982.

[12] D. Uriarte, C. Domini, M. Garrido, New carbon dots based on glycerol and urea and its application in the determination of tetracycline in urine samples, Talanta 201 (2019), pp. 143–148.

[13] X. Zhang, Y. Ren, Z. Ji, J. Fan, Sensitive detection of amoxicillin in aqueous solution with novel fluorescent probes containing boron-doped carbon quantum dots, Journal of Molecular Liquids 311 (2020), Article 113278.
